# Supplementary material for: Animal health perceptions and challenges among smallholder farmers around Kaziranga National Park, Assam, India: A study using participatory epidemiological techniques
Source: PLoS One. 2020 Sep 24;15(9):e0237902. doi: 10.1371/journal.pone.0237902 (PMC7513994; doi:10.1371/journal.pone.0237902)
Supplement: S1 File — (DOCX) [file pone.0237902.s001.docx]

# S1 File

### ***Aide-mémoire* for in-depth interviews**

Village: Date: Time: Interviewer: Translator:

Location/ who else is present / what else is happening?

Name: Male/ female: Age:

**Your family and household**

Who lives in your house? Can we meet them please? What do they do? What do you want to do when grown up?

How is your family/ household supported? (food, money, rent, etc).

What animals does your family/ household have? Can we see your animals please? Why do you keep them? How do animals help to support your household?

What is a ‘good’ animal? How do you know?

How do you know if an animal is health? How do you know if an animal is unhealthy?

What diseases/ problems are important for your animals?

Can you teach me how you recognise the disease? Treat? Prevent?

How often do these problems occur?

What things limit how much your animals give/ provide for your household?

**Young Animals**

Where do you keep your calves? Can we see please?

How many of your cows/ buffs/ goats had a baby in the last year?

Where are those baby animals now?

When a calf is born, can you teach me about the first day of its life?

(When should it first drink milk (colostrum)? How much? What can you do to help it to be healthy? Who helps it?)

How much milk does an older calf drink and how often does it get it? Can you teach me how you do this? Who is responsible for this?

Do your calves have any food apart from milk? Water? Where? How often? Who’s job?

How much milk does your family get each day from your animals? What do you do with it? When do you first milk a cow/ buff /goat for drinking?

**Adult Animals**

What do you do to help your animals? Can you teach me this?

Can you show me where your animals rest in the day? At night?

How do your animals get food? Who (family member) is responsible for this? Can we meet them please? How much land do you have? Can we see it? Where do your animals drink? Can you show me? How often?

Did you vaccinate your animals last year? Why / why not?

Do you de-worm animals? How? Why/ why not?

**Animal health education**

Is there a vet doctor/ Gau Sewek who can treat your animals? What would you like the vet doctor to know more about?

Would you like to learn more about animal health? Why?

What problems are there for people trying to learn about animal health in your village? Why? Which ways of learning about animal health would be good for you (village meetings, classes, leaflets, radio broadcasts, internet information, etc)?

What changes could make your farm to more productive/ better?

What do you think is the future of this farm?

Who will you pass your knowledge of farming on to?
