# Supplementary material for: Animal health perceptions and challenges among smallholder farmers around Kaziranga National Park, Assam, India: A study using participatory epidemiological techniques
Source: PLoS One. 2020 Sep 24;15(9):e0237902. doi: 10.1371/journal.pone.0237902 (PMC7513994; doi:10.1371/journal.pone.0237902)
Supplement: S2 File — (DOCX) [file pone.0237902.s002.docx]

# S2 File

### **Disease descriptors and their frequency of use**

The key features of the conditions discussed by participants are summarised in Supplementary Table 2 below, and the number of highly similar descriptions by meeting groups and interviewees listed. Descriptions of conditions that participants discussed, but decided not to rank, are also included.

**Supplementary Table 2: Frequency of terms used to describe animal health challenges/ conditions by meeting groups and interviewees.**

| **Condition** | **Description** | **Number of similar descriptions** | |
| --- | --- | --- | --- |
|  |  | **Meetings** | **Interviews** |
| **Chaboka / Kurra phata** | Wound comes in interdigital space, hoof separates from coronary band and maggots come. Animal is lame. | **9** | **17** |
|  | Wounds come in mouth and excess salivation occurs. Sometimes animal can’t eat or drink. | **6** | **13** |
|  | Maggots come to wounds | **7** | **9** |
|  | Abortion occurs occasionally | **1** | **3** |
|  | Spreads rapidly | **4** | **17** |
|  | Doesn’t affect every animal every year | **1** | **5** |
|  | Occurs during rainy season | **3** |  |
|  | Occurs after flood | **2** | **12** |
|  | Occurs at any time of year | **1** | **11** |
|  | Cattle and buffalo affected | **6** | **17** |
|  | Goats affected | **5** | **3** |
|  | Pigs affected | **1** |  |
|  | Vaccination reduces/ controls problem | **4** | **13** |
| **Jor** | Lays down, won’t eat, body/ears feel hot | **3** | **3** |
|  | Cows affected | **2** | **3** |
|  | Goats affected |  | **1** |
|  | Pigs affected | **1** |  |
| **Pelu** | See worms in dung |  | **3** |
|  | Lose weight | **3** | **1** |
|  | Diarrheoa | **3** | **1** |
|  | Goats affected | **3** | **3** |
|  | Calves affected | **2** |  |
|  | Young goats worse affected | **2** | **1** |
|  | May die | **1** | **1** |
| **Pet phulla** | The belly is swollen (distended) | **7** | **13** |
|  | The animal stops eating and drinking and does not pass urine or dung | **3** | **9** |
|  | Cattle affected | **7** | **13** |
|  | Goats affected | **4** | **4** |
|  | Occurs at grazing or in shed | **-** | **6** |
|  | Occurs at grazing | **1** | **3** |
|  | Occurs after flood | **3** | **3** |
|  | Occurs in winter | **3** | **2** |
|  | Occurs at any time | **-** | **4** |
|  | Usually one or two animals affected at a time | **1** | **13** |
|  | Usually die | **3** | **1** |
|  | May die without treatment | **1** | **9** |
|  | Usually survive | **-** | **1** |
| **Sikora** | Ticks | **1** | **3** |
|  | Weakens animal if many present | **-** | **3** |
|  | Worst in winter | **-** | **2** |
|  | Worst in hot season | **-** | **1** |
| **Hagoni** | Dark green / black diarrhoea | **4** | **10** |
|  | Occurs on new grass after flood | **7** | **10** |
|  | Many animals affected | **4** | **10** |
|  | Young animals worst affected | **2** | **1** |
|  | All ages affected equally | **4** | **5** |
|  | Goats affected | **6** | **10** |
|  | Calves affected | **3** | **5** |
|  | Pigs affected | **1** | **-** |
|  | All / most die | **4** | **5** |
|  | Some die | **3** | **3** |
|  | Most survive | **-** | **1** |
| **Anthrax** | Cattle and buffalos stop grazing. Fat belly occurs and blood from nose | **1** | **1** |
|  | Outbreaks affect domestic and wild animals | **1** | **1** |
|  | Always die with a few hours (or faster) | **1** | **1** |
| **Letekua** | Eye turns white | **3** | **6** |
|  | Discharge from eye | **3** | **4** |
|  | Goats affected | **2** | **5** |
|  | Cattle affected | **1** | **2** |
|  | One eye affected at a time | **1** |  |
|  | May affect one or both eyes | **2** | **5** |
|  | One animal affected at a time | **2** | **2** |
|  | Can be more than one animal affected | **-** | **3** |
|  | May go blind, especially if not treated | **2** | **5** |
|  | Occurs in dry season | **-** | **2** |
|  | Can occur at any time of year | **-** | **2** |
| **Papora dhora** | White, flaky, raised, thick skin | **7** | **8** |
|  | Hair falls out | **4** | **8** |
|  | Itchy | **3** | **5** |
|  | Spreads over body, can affect whole body | **-** | **7** |
|  | Goats affected | **6** | **8** |
|  | Dogs affected | **2** | **2** |
|  | Calves affected | **-** | **2** |
|  | People affected | **-** | **2** |
|  | All ages of animal affected | **1** | **2** |
|  | Spreads between animals | **4** | **7** |
|  | Worse in dry season | **2** | **5** |
|  | Occurs at any time of year | **1** | **1** |
|  | Become well with treatment | **1** | **7** |
|  | Never die | **2** | **2** |
|  | May die if not treated |  | **2** |
|  | Become thin and weak | **2** | **3** |
| **Gol phulla / bhekulia/ Dhoka dingra /Dingi fulla/**  **(Condition 1)** | Swelling under chin, salivation, breathing problems. Dies. Cattle disease. | **4** | **5** |
|  | Occurs after flood | **1** | **-** |
|  | Occurs in winter | **1** | **1** |
|  | Vaccination prevents | **-** | **2** |
| **Gol phulla / bhekulia**  **(Condition 2)** | Swelling under jaw, lose weight, don’t die.  Cattle and goats affected. | **1** | **4** |
|  | Swelling under jaw, lose weight, die.  Cattle and goats affected. | **1** | **2** |
|  | Occurs after flood | **2** | **2** |
|  | Occurs before flood |  | **1** |
| **Sagolay bohonta / basanta / Kucheswan** | Hard raised lumps on body | **7** | ***** |
|  | Lumps may burst/ ulcerate | **3** | ***** |
|  | Hair falls out around lumps | **1** | ***** |
|  | Discharge from nose / sores around nostrils | **6** | ***** |
|  | Eyes swollen and discharge | **4** | ***** |
|  | Cough and fast breathing | **3** | ***** |
|  | Shivering and fever | **3** | ***** |
|  | Stops eating / sores in/ around mouth | **5** | ***** |
|  | Lame | **3** | ***** |
|  | Die | **7** | ***** |
|  | Spreads between animals | **6** | ***** |
|  | Occurs after flood | **2** | ***** |
|  | Goats affected | **7** | ***** |
| **Ranikhet / Murgi- julka- loga / Hal- julka- loga** | Duck or chicken appears sleepy | **6** | **2** |
|  | Head down and neck turned round | **6** | **2** |
|  | Breathing problems | **3** | **1** |
|  | Wings spread | **3** | **1** |
|  | White dung | **5** | **2** |
|  | Die very quickly (duck= hours, chicken< 2 days) | **9** | **2** |
|  | Duck: occurs after flood | **3** | **2** |
|  | Chicken occurs when cotton seed tree flowers | **5** | **1** |
|  | Spreads very fast | **8** | **2** |
|  | All birds in household died | **-** | **2** |
|  | When disease comes most birds in village die | **4** | **1** |
| **Pani howa** | Cow. Eye waters. | **1** | **-** |
| **Murigoni** | Circling goat | **2** | **2** |
|  | Head tilt to one side and goat turns towards it | **2** | **2** |
|  | One animal at a time affected | **2** | **2** |
|  | Adult goats affected | **2** | **2** |
|  | Goat dies | **2** | **2** |
| **Goat hoof cracks** | Many goats affected. Upper surface cracks, difficult to walk. Occurs at any time | **1** | **-** |
| **Kandh Singha** | Hump sore from yoke on draft oxen | **2** | **2** |
|  | Can’t work properly | **2** | **2** |
|  | Flies and maggots come | **1** | **2** |
| **Hafonee** | Fever, shaking, anorexia | **3** | **-** |
|  | Cows affected | **2** | **-** |
|  | Any time of year | **1** | **-** |
|  | A few animals affected at a time | **1** | **-** |
|  | May die | **1** | **-** |
| **Poor growth goat** | Goats eat but do not thrive | **1** | **-** |
| **Bat- hera** | Teat blocked goat. One side of udder hard. Babies can’t feed (Occurs in many goats) | **1** | **-** |
| **Moh- bis- oni** | Leg, legs or whole body swells rapidly | **2** | **2** |
|  | Crackling sound under skin when touched |  | **2** |
|  | Skin feels wet |  | **1** |
|  | One animal at a time affected |  | **1** |
|  | Animal dies in 12 – 24 hours | **2** | **2** |
|  | Cattle of any age affected | **2** | **2** |
|  | Vaccination prevents | **1** | **3** |
| **Okoni** | Flea or lice |  | **1** |
| **Geva- ghuti (dhena)** | Tongue swells (cow), one animal at a time, doesn’t spread between animals. Can’t eat. Dies if untreated (puncture tongue) | **1** | **-** |
| **How- phora** | White jelly in cow dung. Animal is weak. Older cow affected. Recovers in 2 – 3 days | **1** | **-** |

***Sagolay Bohonta outbreak occurred in the villages where the interviews were carried out after the interview process was completed. The condition was previously unknown in these villages.**

**- Not mentioned, or condition not discussed by participants**

**Chaboka/ Kurra patta**

*Chaboka* is a specific description of foot and mouth disease (FMD). *Kurra patta*, meaning broken hoof also indicates FMD. A small number of respondents thought these were different conditions- *chaboka* a mouth disease and *kurra phatta* a foot problem, but the vast majority of participants recognised that the signs were linked in a single disease.

*“Saliva drips, the tongue is infected, there are lesions on the mouth, lips and tongue. Many animals are affected, and it spreads between them. Animals suffer a lot but then become well. Very occasionally abortion occurs. This occurs after flood.”*

Interviewee 3, female 30

“*Chaboka” or Kura- patta: the cow can’t walk, a wound runs around top of hoof, blood comes, then flies and maggots. Sometimes animals don’t eat and saliva drips. Infection may also occur inside the mouth and nose. Occurs after flood, spreads fast and can affect every animal. If an animal catches one year, it usually doesn’t catch next year.*

Interviewee 7, male 42

*“Chaboka- comes after flood, it is also called Kura- Patta (Broken hoof), it’s the same problem: This is our main problem. A wound comes in the feet- blood comes out – maggots come. The animal stops eating, tongue infection can occur. Fever also. It spreads from one to many. Not always every year in every animal. … With good treatment full recovery happens in 3- 4 months. Oxen cannot work when they are affected, and this can be a big problem for us.”*

Interviewee 11, male 58

**Ranikhet / Murgi- julka- loga / Hal- julka- loga**

These terms refer specifically to Newcastle Disease, though not all respondents were aware of a name for this condition.

*“No name disease, it affects the ducks- saliva comes from their mouths, sometimes all* (of the) *flock dies. It spreads quickly from one to another. They stop walking, sleep a lot, then they die. They never survive, die in less than 24 hours... The disease comes after the flood”*

Interviewee 12, male 35

*“Hagoni* (dysentery) *of ducks and chicken. Their dung turns white, and they die. They are sleepy. There is no treatment. Nowadays this disease can come at any time of year, it used to be just in the dry season. This year the disease came after the flood. All our* (household’s) *ducks and chickens died.”*

Interviewee 1, male 45

*“Sleepy chicken disease, Ranikhet, comes when the silk cotton trees are in flower. Then chickens are affected. Ducks are affected more after the flood.”*

Female attendee, Meeting 10

(Silk cotton trees flower in February and March producing large quantities of seeds which attracts many wild birds).

**Gol phulla / bhekulia / (Dhoka dingra, dingi phulla,)**

These terms encompass conditions involving swelling of the neck or throat, but do not appear to be used consistently from village to village or person to person. It seems likely that this description includes haemorrhagic septicaemia and chronic fasciolosis. It is also possible that acute pneumonia, allergy or tooth root abscess are included by some respondents. Some respondents used more than one of these terms to describe different conditions, others used more than one term to describe the same condition.

*“Gol phulla - cows and oxen are affected, they die. In 24 – 48 hours they die. There is a lot of saliva and hard breathing, the animal will not eat. Jerseys and younger animals are the worst affected… occurs in wintertime mostly”*

Female attendee, meeting 7

*“Dhoka- Dhingira- the outside of the neck swells, the cow stops drinking and urinating. The animal soon dies. Occurs during winter season, usually between midnight and dawn. Only happens to strong cow and ox.”*

Interviewee 13, male 52

*“Gol- phulla is also called “Bhekulia. Goats also affected as well as cows. Swelling of the neck- first the chin, then running down* (ventral) *throat, and the animal gets weaker day by day, stops eating. After 12- 13 days- the animal becomes well again.”*

Interviewee 7, male 42

*“Dingi- Phulla or Gol-Phulla means neck swelling. One or two animals are affected at a time, it occurs after the flood. The animals die every time after three to four days. As well as the swelling under chin there is also dysentery- very watery.”*

Interviewee 9, male 43

*“Dhingi Fulla is swelling under chin, it spreads down* (ventrally*) a bit. Cow and goat are both affected.* (It occurs during the) *Hot season. One animal is affected at a time. No other signs. They become well in 2 – 3 days, but get thin and eat less.”*

Interviewee 14, female 34

**Hagoni**

*Hagoni* represents a range of conditions characterised diarrhoea and dysentery. Respondents’ descriptions include per-acute disease of goats on new grass after the flood (which, from descriptions, may sometimes be clostridial in origin?), neonatal diarrhoea, and diarrhoea of dietary origin. Parasitic gastro-enteritis leading to loose motions is also be captured within this description, as well as other aetiologies.

*“Hagoni is goat dysentery… After flood when fresh grass comes, every year, many are affected… the whole village. Goats die in 2-3 days, young and old are both affected.”*

Interviewee 1, male 49

*“Goat dysentery occurs after eating new grass after flood. Most die. It happens every year.”*

Interviewee 2, female 44

*“Hagoni is loose motions, goat or calf are affected, mostly young animals, usually occurs after flood. If the dysentery is bloody the animal will die, otherwise they may survive, but sometimes die.”*

Interviewee 9, male 43

*“In goats hagoni is common after the flood, any goat can be affected… there is dark diarrhoea… most die. In calves hagoni occurs after the flood, usually when the calf is 2- 3 months old and it starts to graze.”*

Male attendee, meeting 8

*“Young goats aged 6 months – 1 year are most affected. Most die. If doctor treats them- they can get well. Usually they die in 2 – 4 days... Black watery dung, no blood, it stinks… Cow, mostly older animals are affected one by one, they get very weak. Doctor can them treat and they get well, otherwise they die. The same disease spreads to the calf.”*

Male attendees, meeting 9

**Pet phulla**

*Pet phulla* indicates conditions characterised by abdominal distension. Gas bloat would appear to be one of the more common conditions described, but sudden death followed by rapid post mortem bloating may also sometimes captured by this term.

*“Pet phulla means fat belly. The animal swells and suffers breathlessness. Dies without treatment in one to one and a half hours… Cow, goat and calf can all be affected. One animal at a time. Pet phulla occurs at any time… in the cow shed and out at grazing.”*

Interviewee 5, female 37

*“Pet- phulla is a gas problem… occurs after flood, all animals may be affected. Out at the paddy or in the shed... Die unless treated.”*

Interviewee 6, male 49

*“I had a one-year old calf* (that suffered pet phulla)*, she was very healthy. One day she ate breakfast, then stopped eating and went out to the paddy. I found dead a few hours later, her belly was fat (distended) both sides.”*

Interviewee 1, male 49

*“Pet- Phulla. The animal has a big belly and harsh breathing. They fall down, lift their tail and strain to pass dung- it gets worse with each attempt. Occurs in cows, not calves, out at the paddy or in the shed.”*

Interviewee 15, female 45

*“Pet phulla affects cows and goats. It happens out in the paddy fields, but we notice it once we bring the animals home. It is caused by eating new grass covered in mud* (from the flood*) … They die in two hours, or sometimes 1 – 2 days. Call the (veterinary) Doctor… sometimes they live if treated.”*

Male attendee, meeting 6

**Sagolay bohonta**

*Sagolay is the Assamese word for a goat, bohonta* or *basanta* indicate a pox or similar skin condition. While the term *bohonta* or *basanta* is used generally to indicate various conditions characterised by skin lesions, it is also used specifically to indicate a disease that can be definitively diagnosed as goat pox. An outbreak of goat pox occurred during the data gathering process, subsequent to the completion of the in-depth interviewing. At time of undertaking village meetings, two villages had never experienced the disease, six villages were experiencing the disease for the first time, and two villages experienced the disease regularly. In those village that had not previously encountered the disease, the clinical situation was that of a naïve population encountering the disease (9).

*“Goats are affected… Lumps come over the goat’s whole body, may burst later. Their eyes swollen and water. Water comes from nose and mouth, cough (soft cough) and fast breathing, the goat does not eat, it picks its feet up as it hurts to walk. They die in one week…. Bohonta has killed all the goats in this village... This is the first time we have had bohonta here”*

Female attendees, meeting 6

*“Lumps come all over the body, there are sores around the mouth and nose, and discharges come. All affected goats die… Here* (in this village*) there are over 120 households, nearly all our goats died… We had never seen this disease before.”*

Attendees, meeting 9

*“Hard raised lumps come all over body. The goat has fever. Then hair falls* (out) *and the lumps may open. Discharge comes from the nose and eyes. And a cough. Spreads between animals. Goats die… Several farms are affected every year… Sometimes we buy in goats from outside places.”*

Male attendees, meeting 4

**Papora dhora**

**This term specifically describes trhe clinical condition seen when an animal suffers from sarcoptic mange.**

*“Papora- dhora is goat skin disease…* (the goat) *rubs it’s body against anything, it is very itchy, hair falls, the skin is rough and a white colour with some boils. (Papora dhora) Spreads and can affect all goats, it starts after the flood and goes on after that. Affected goats can get thin. Papora dhora affects dogs also. May affect calves too. Animals become well later, but it takes time.”*

Interviewee 16, female, 36

*“Papora- dhora occurs at any time of year, it starts on one goat and spreads between animals. Starts on one area of the goat and spreads. Whole body is dry, hair falls out in places, the skin below is white, thickened, dry flaky with many deep wrinkles.”*

Interviewee 8, female, 25

Reviewing these conditions considered of particular importance by participants, it can be seen that the seven Assamese terms encompass four specific infectious viral disease plagues, two parasitic diseases, and three primary symptoms which can encompass a variety of aetiologies.
