# Supplementary material for: Animal health perceptions and challenges among smallholder farmers around Kaziranga National Park, Assam, India: A study using participatory epidemiological techniques
Source: PLoS One. 2020 Sep 24;15(9):e0237902. doi: 10.1371/journal.pone.0237902 (PMC7513994; doi:10.1371/journal.pone.0237902)
Supplement: S3 File — (DOCX) [file pone.0237902.s003.docx]

# **S3 File**

Details of village meetings, attendance, animal species kept, disease descriptors, breakdown of proportional piling exercises, and interview notes.

**Kaziranga Participatory Epidemiology Results Summary**

| Village | Date | Attendance | | |
| --- | --- | --- | --- | --- |
|  |  | Male | Female | Total |
| A | 21/3/17 | 6 | 17 | 23 |
| B | 12/10/17 | 8 | 14 | 22 |
| C | 12/10/17 | 1 | 21 | 22 |
| D | 13/10/17 | 17 | 0 | 17 |
| E | 12/2/18 | 11 | 14 | 25 |
| F | 13/2/18 | 11 | 0 | 11 |
| G | 15/2/18 | 11 | 5 | 16 |
| H | 16/2/18 | 13 | 2 | 15 |
| I | 17/2/18 | 12 | 7 | 19 |
| J | 17/2/18 | 7 | 7 | 14 |
| **Total** |  | **97 (53%)** | **87 (47%)** | **184 (Av size 18)** |

| Village | Cow | Oxen | Goat | Pig | Buffalo | Chicken | Duck | Pigeon | Comments |
| --- | --- | --- | --- | --- | --- | --- | --- | --- | --- |
| A | Y | Y | Y | N | N | Y | Y | Y |  |
| B | Y | N | Y | Y | N | Y | Y | Y |  |
| C | Y | Y | Y | Y | Y | Y | Y | N |  |
| D | Y | Y | Y | Y | N | Y | Y | N |  |
| E | Y | Y | Y | N | Y | Y | Y | Y |  |
| F | Y | Y | Y | Y | Y | Y | Y | Y |  |
| G | Y | Y | Y | Y | N | Y | Y | Y |  |
| H | Y | Y | Y | Y | N | Y | Y | Y |  |
| I | Y | N | Y | N | Y | Y | Y | Y |  |
| J | Y | N | Y | N | Y | Y | Y | Y |  |
| Total | 10 | 7 | 10 | 6 | 5 | 10 | 10 | 8 |  |

| **Condition** | **Proportional piling score (-/100) assigned by each village group** | | | | | | | | | |
| --- | --- | --- | --- | --- | --- | --- | --- | --- | --- | --- |
|  | **Village** | | | | | | | | | |
|  | **A** | **B** | **C*** | **D** | **E** | **F*** | **G** | **H*** | **I** | **J** |
| **Chaboka / Kurra phata** | **17** | 11 | 8 | 10 | 15 | 15 | 20 | 16 | 5 | **16** |
| **Jor** | 13 |  | 13 |  |  |  |  |  |  |  |
| **Pelu** | 10 |  |  |  |  |  | 15 |  |  |  |
| **Pet phulla** | 9 | 8 | 14 |  | 14 |  |  | 8 |  | 9 |
| **Sikora** | 9 | 4 |  |  |  |  |  |  |  | 6 |
| **Hagoni** | 7 |  | 15 | **23** |  | 22 |  | 11 | 15 | 7 |
| **Anthrax** | 6 | 12 |  |  |  |  |  |  |  |  |
| **Letekua** | 6 |  |  |  |  |  |  | 9 |  |  |
| **Phapora dhora** | 5 | 19 |  | 13 | 17 | 25 | 10 |  |  | 7 |
| **Dhoka dingra** | 5 |  |  |  |  |  |  |  |  |  |
| **Bohonta / basanta** | 4 |  |  |  |  |  |  |  |  |  |
| **Ranikhet / Murgi- julka- loga / Hal** |  | 20 | 19 | 9 | 15 | 14 | 7 | 9 | 15 | 14 |
| **Gol phulla / bhekulia** |  | 5 | 2 | 10 | **21** |  | **26** | **27** | **20** | 11 |
| **Pani howa** |  |  |  | 10 |  |  |  |  |  |  |
| **Murigoni** |  |  |  | 7 |  |  |  |  |  |  |
| **Goat hoof cracks** |  |  |  |  | 14 |  |  |  |  |  |
| **Sagolay bohonta** |  | **22** | **19** | 18 | 5 |  |  | 15 | **20** | 14 |
| **Kandh Singha** |  |  |  |  |  | 24 |  |  | 5 |  |
| **Hafonee** |  |  |  |  |  |  | 13 |  |  |  |
| **Poor growth goat** |  |  |  |  |  |  | 4 |  |  |  |
| **Bat- hera** |  |  |  |  |  |  |  | 5 |  |  |
| **Moh- bis- oni (BQ)** |  |  |  |  |  |  |  |  | 5 | 10 |
| **Shivering cow** |  |  |  |  |  |  |  |  |  |  |
| **Okoni** |  |  |  |  |  |  |  |  |  |  |
|  |  |  |  |  |  |  |  |  |  |  |

***Village C wanted to rank conditions initially, then re- ordered them following the piling exercise**

****Village D did not want to include Ranikhet as thet identified themselves as cattle and goat farmers. However when aked to re-rank the conditions in terms of financial impact they placed sagolay bohonta in position 1 and ranikhet in position 2.**

*****Village F**: **Did the piling exercise based on prevalence, then when they saw the results completely re-ordered based on disease impact, but did not wish to repeat piling exercise.**

******Village H: Did the piling exercise, discussed the results, then re-did the piling exercise from the start.**

Other conditions discussed:

Geva- ghuti: cow tongue swells, doesn’t spread. Can’t eat, dies if untreated. Puncture tongue and put alum and dimorh (herb)

Murigoni: Pregnant goats (and other adults) after flood, May die.

Aged 1-2 years. Always die. Either takes 2-3 days or 7-8 days

Hagoni: Last year 700- 800 goats died in a diarrhoea outbreak in this village

Ranikhet:

Okoni

Anthrax: Occurred only once, most cattle died here

Murgi okoni: Also bites people

HS: Discussed by letter, villages said used to be a big problem, now vaccinate and no problem.

BQ: Discussed by letter, villages said used to be a big problem, now vaccinate and no problem.

Mackie- puk

Cow all hair falls: 2 cows affected, one died

Placenta doesn’t fall: call Dr

Cow coughs at night: sell this cow

Cow swollen leg (one)

Cow not showing oestrus

Shivering cow (same as Hafonee?)

| **Condition** | **Rank (-/8) assigned by each village group** | | | | | | | | | |
| --- | --- | --- | --- | --- | --- | --- | --- | --- | --- | --- |
|  | **Village** | | | | | | | | | |
|  | **A** | **B** | **C** | **D** | **E** | **F*** | **G** | **H** | **I** | **J** |
| **Chaboka / Kurra phata** | **1** | 5 | 6 | 4 | 3 | 5 | 2 | 2 | 5 | **1** |
| **Jor** | 2 |  | 5 |  |  |  |  |  |  |  |
| **Pelu** | 3 |  |  |  |  |  | 3 |  |  |  |
| **Pet phulla** | 4 | 6 | 4 |  | 5 | 3 |  | 7 |  | 6 |
| **Sikora** | 4 | 8 |  |  |  |  |  |  |  |  |
| **Hagoni** | 6 | 4 | 3 | **1** |  | 2 |  | 4 | 3 | 7 |
| **Anthrax** | 7 |  |  |  |  |  |  |  |  |  |
| **Letekua** | 7 |  |  |  |  |  |  | 5 |  |  |
| **Phapora dhora** |  | 3 |  | 3 | 2 | 4 | 5 |  |  | 7 |
| **Dhoka dingra** |  |  |  |  |  |  |  |  |  |  |
| **Bohonta / basanta** |  |  |  |  |  |  |  |  |  |  |
| **Ranikhet / Murgi- julka- loga / Hal** |  | 2 | 2 | 7 | 3 | **1** | 6 | 5 | 3 | 2 |
| **Gol phulla / bhekulia** |  | 7 | 7 | 4 | **1** |  | **1** | **1** | **1** | 4 |
| **Pani howa (HS?)** |  |  |  | 4 |  |  |  |  |  |  |
| **Murigoni** |  |  |  | 8 |  |  |  |  |  |  |
| **Goat hoof cracks** |  |  |  |  | 5 |  |  |  |  |  |
| **Sagolay bohonta** |  | **1** | **1** | 2 | 7 |  |  | 3 | **1** | 2 |
| **Kandh Singha** |  |  |  |  |  | 6 |  |  | 5 |  |
| **Hafonee** |  |  |  |  |  |  | 4 |  |  |  |
| **Poor growth goat** |  |  |  |  |  |  | 7 |  |  |  |
| **Bat- hera** |  |  |  |  |  |  |  | 8 |  |  |
| **Moh- bis- oni (BQ)** |  |  |  |  |  |  |  |  | 5 | 5 |
| **Shivering cow** |  |  |  |  |  |  |  |  |  |  |
| **Okoni** |  |  |  |  |  |  |  |  |  |  |

What is Gol phulla?

Village:

1. Dhoka dhingra?
2. Cow- swelling under chin, cough also, can’t eat. Dies in 7 – 14 days. Occurs in August- September, doesn’t spread between animals.
3. Swelling below jaw, don’t die, last 1 -2 weeks.
4. Swelling under jaw, occurs after flood, may be several animal but occurs one by one (does spread from one animal to another). Animal gets thin. Don’t die, doctor treats.
5. After flood swelling occurs, diarrhoea, can’t eat properly, slow disease, loss of condition, dies.
6. –
7. **Cow dies in 24 - 48 hours. Salivates, can’t eat, hard breathing. Younger jerseys particularly affected. Winter time mostly.**
8. Cow, also calf. Slow disease, weak animals (10%). Give tablet- gets better, may die if very weak. (deworm- gets better? – same problem?)
9. **Cow and calf affected. Occurs at any time. Few survive- die in a few days. Becomes weak first, cough occurs. Hard breathing? – don’t know.**
10. **Cow- dies (within 15 days?)- spreads, fever, hard breathing.**

| **Condition** | **Number of times ranked** |
| --- | --- |
| **Chaboka / Kurra phata** | **10** |
| **Jor** | **2** |
| **Pelu** | **2** |
| **Pet phulla** | **7** |
| **Sikora** | **2** |
| **Hagoni** | **8** |
| **Anthrax** | **1** |
| **Letekua** | **2** |
| **Phapora dhora** | **6** |
| **Bohonta / basanta** | **1** |
| **Ranikhet / Murgi- julka- loga / Hal** | **9** |
| **Gol phulla / bhekulia / Dhoka dingra** | **8** |
| **Pani howa (HS?)** | **1** |
| **Murigoni** | **1** |
| **Goat hoof cracks** | **1** |
| **Sagolay bohonta** | **7** |
| **Kandh Singha** | **2** |
| **Hafonee** | **1** |
| **Geva- ghuti (Dhenia)** | **1** |
| **Poor growth goat** | **1** |
| **Bat- hera** | **1** |
| **Moh- bis- oni (BQ)** | **2** |
| **Shivering cow** |  |
| **Okoni** |  |

**Chaboka / Kurra phata**

| **Village / rank** | | **Description** |
| --- | --- | --- |
| A | **1** |  |
| B | **5** | Occurs in June and July- during rainy season. Wound occurs in interdigital space, hoof separates and then maggots come. No mouth problem, abortion occasionally occurs.  Very seasonal, does not affect every animal every year. Usually cattle, rarely goats. If pigs are affected they die |
| C | **6** | Affects cow and goat. Lame, salivation, animal can’t work |
| D | **4** |  |
| E | **3** | **After flood, cow, buff, goats affected. Maggots come in foot, bad smell, No mouth signs in cow but do occur in goats. Goat can die, but not cow. Reduced milk production? - don’t know. Abortion? – don’t know.Use crop pesticides on maggots.** |
| F | **5** | **Cow, buffalo, bullock, goat. Not pig.**  **Maggots come inside foot- petrol or crop insecticides used.**  **We use vaccine programmes when offered**  **Mouth; can’t eat or drink- animal suffers a lot. Rub mouth with bamboo leaves.** |
| G | **2** | **Cow, goat, ox. Not pig. Occurs during flood. Lameness, spreads** |
| H | **2** | **Buff, goat, cow, occurs at any time of year. Wound between toes, can spread round hoof. Salivation also. Forest department don’t come regularly for vaccination. Some farmers buy vaccine privately. If vaccine is given there is no problem.** |
| I | **5** | **Cow mostly, also goat, and occasionally buffalo.**  **Now occurrence is very less because of vaccination** |
| J | **1** | **Cow goat, spreads rapidly, occurs after flood.**  **Vaccine now used, so problem is very much less.** |

**Jor**

| **Village / rank** | | **Description** |
| --- | --- | --- |
| A | 2 |  |
| C | 5 | loss of appetite- pigs |

**Pelu**

| **Village / rank** | | **Description** |
| --- | --- | --- |
| A | 3 |  |
| G | **3** | **Goats, aged 6 months – 1 year** |

**Pet Fulla**

| **Village /rank** | | **Description** |
| --- | --- | --- |
| A | 4 |  |
| B | 6 | Stomach swells in 24 hours, animal doesn’t eat. No dung, then urine stops also. Salivate, die in next 12 hours. Increased respiratory rate, no neck swelling. Occurs to animals one by one, doesn’t spread. Affects cow, ox, heifer, never goats. Happens in field, particularly January and Feburary. |
| C | 4 | Affects cows. Belly swells. May die or become well |
| E | **5** | **Goat: die in 3 days, always die. Occurs after flood when new grass comes.**  **Cow: starts with gol phulla, Winter season last year. One case at a time but may affect many animals. Die in 24 hours.** |
| F | **3** | **Goat: happens in paddy fields but noticed when animal comes home. New grass covered in mud. Die 2.5 hours, or 1 – 2 days. No home treatment, sometimes live if treated by doctor.**  **Cow:** |
| H | **7** | **Cow, belly swollen, old and young, one animal at a time. Occurs in winter, never summer or flood.** |
| J | **6** | **Cow. Occurs at any time of year, young and old. Die.**  **Goat:** |

**Sikora**

| **Village / rank** | | **Description** |
| --- | --- | --- |
| A | 4 |  |
| B | **8** |  |

**Hagoni**

| **Village /rank** | | **Description** |
| --- | --- | --- |
| A | 6 |  |
| B | 5 | Diarrhoea, usually black. Young animals- cow and goat, baby calf. Treat with black salt. Sometimes die.  **Last year 700 – 800 goats died of a diarrhoea type illness.** |
| C | 3 | Cow, calf, goat, pig |
| D | **1** |  |
| F | **2** | **Cow: mostly older animal, one by one, get very weak. Doctor can treat and they get well, otherwise die. Same disease spreads to calf.**  **Goat: Young goats 6 months – 1 year. Most die. If doctor treats- can get well. Die in 2 – 4 days. Black watery dung, no blood, stinks.** |
| H | **4** | **Dark diarrhoea. Goats, any age, after flood. Die.**  **+/- worms, but villagers think this is a different problem.** |
| I | **3** | **Goats: common after flood, any goat can be affected, most die.**  **Calf: occurs after flood, when calf is 2 – 3 months old and starts to graze.** |
| J | **7** | **Goat- new grass comes after flood. All ages affected, dark green diarrhoea. Die. Doctor may treat effectively. Other man says doctor medicine not effective** |

**Anthrax**

| **Village / rank** | | **Description** |
| --- | --- | --- |
| A | 7 | Outbreak in park several years ago. Forest department dug huge pit to dispose of bodies |

**Letukua**

| **Village / rank** | | **Description** |
| --- | --- | --- |
| A | 7 |  |
| H | **5** | **Goat white eye. Become well if treated. But if goes blind in both eyes, dies from an accident.** |

**Phapora Dhora**

| **Village / rank** | | **Description** |
| --- | --- | --- |
| A | 9 |  |
| B | **3** | Goats, whole body, hair falls, skin splits. Itchy, spreads between goats. Worse in winter. |
| D | **3** | Goat. Skin rise. |
| E | **2** | **Goats, all times of year, spreads, never die. Treat with injection “ numec”, one injection, sometimes two.** |
| F | **4** | **Goats, winter time especially, dogs also, weaken, don’t thrive, cost of medicine.** |
| G | **5** | **Goats only. White crust. Skin rise** |
| J |  | **Affects goats. Dogs also. Spreads. White risen skin.** |

**Raniket aka Murgi (or Hal) juluka loga**

| **Village / rank** | | **Description** |
| --- | --- | --- |
| B | 2 | Chicken- white dung, comb turns black, continuous breathing sound *(snicking?)*, drink water fast, don’t eat. Die in 3-4 days, disease spreads very fast. |
| C | **2** | Duck and chicken, white dung, water from eye, head down, sleepy, die in 2-3 days. Disease spreads fast between birds. |
| D | **-** | These participants (all men) didn’t want to include Ranikhet in the piling exercise as they largely see themselves as cattle and goat farmers. One men who had suffered high losses did, but he was over ruled by the group. In some households poultry is considered a woman’s responsibility. |
| E | **3** | **“Sleeping disease”- always die, ducks die in 30 minutes. Duck disease occurs after flood, chicken- anytime. Water from nose, weight loss. One mean had 85 ducks die. When disease comes the whole village is affected and all die, maybe 1 or 2 survive.** |
| F | **1** | **Duck: spreads wings and dies in 2 hours.**  **Chicken: head down and dies in 2 days.**  **Spreads whole village. Starts when cotton seed tree blooms and seeds fall.** |
| G | **6** | **Aka “Murgi- jupa” , “Bengal tea garden disease”**  **Head turned, wings spread, white diarrhoea, most die (>80%), often within one day from becoming ill.**  **“We find it difficult to compare a chicken problem with a cow or goat problem. Maybe we should give this disease an increased rank.”** |
| H | **5** | **Sleepy chicken. Head down, dies. Occurs in spring (when cotton seed tree seeds fall). Also in ducks around flood time.** |
| I | **3** | **“Murgi – joluka – loga”**  **Bird down, head round, white dung, dies.**  **Spreads fast.**  **Happening now (Silk cotton tree in bloom)** |
| J | **2** | **“Murgi- juluka- loga” (or “Hal”), die, very quick disease. White dung, sleepy, wings spread, head round.**  **Duck: after flood**  **Chicken: when silk cotton tree in flower** |

**Gol phulla / bhekulia / Dhoka- dingra**

| **Village / rank** | | **Description** |
| --- | --- | --- |
| A | 9 |  |
| B | **7** | Cow- swelling under chin, cough also, can’t eat. Dies in 7 – 14 days. Occurs in August- September, doesn’t spread between animals. |
| C | **7** | Swelling below jaw, don’t die, last 1 -2 weeks. |
| D | **5** |  |
| E | **1** | **After flood swelling occurs, diarrhoea, can’t eat properly, slow disease, loss of condition, dies.** |
| G | **1** | **Cow dies in 24 - 48 hours. Salivates, can’t eat, hard breathing. Younger jerseys particularly affected.**  **Winter time mostly.** |
| H | **1** | **Cow, also calf. Slow disease, weak animals (10%). Give tablet- gets better, may die if very weak. (deworm- gets better? – same problem?)** |
| I | **1** | **Cow and calf affected. Occurs at any time. Few survive- die in a few days.**  **Becomes weak first, cough occurs. Hard breathing? – don’t know.**  HS and BQ (said the letters), participants didn’t know local names. Used to cause deaths- hard breathing, now give vaccine- no problem. |
| J | **4** | **Cow- dies (within 15 days?)- spreads, fever, hard breathing.** |

**Pani Howa**

| **Village / rank** | | **Description** |
| --- | --- | --- |
| D | 6 | Eye waters |

**Murigoni**

| **Village / rank** | | **Description** |
| --- | --- | --- |
| D | 7 | Circling goat. Happens occasionally, usually dies |
| B | **10** | Circling goat- die every time, takes either 2-3 days or 7-8 days. 1 goat affected sat a time- usually aged between 1 and 2 years. |

**Goat hoof cracks**

| **Village / rank** | | **Description** |
| --- | --- | --- |
| E | 5 | **Many goats. Upper surface cracks, difficult to walk. Occurs at any time** |
|  |  |  |

**Sagolay Bohonta**

| **Village / rank** | | **Description** |
| --- | --- | --- |
| A |  | Meeting taken prior to first outbreak |
| B | 1 | **Mostly goat, also cow. Shivering and fever- small swellings/ blisters all over body (these burst), animal stops eating, dies. Course of disease takes about two weeks. If doctor gives medicine (2 injections)- animal improves.**  **Bohonta spreads. Occurs after flood season. This year all village affected. Cough and fast breathing, water from eyes. Blisters can be seen from a distance, feel hard. Animals die.**  **80% of village goats dead. All ages affected, some survive, but not many. From people at meeting 61 goats belonging to attendees reported dead.** |
| C | 1 | **Bohonta has kill all (90%) goats in this village. Attendees have lost 114 goats between them. This is the first time we have had bohonta.**  **Lumps over whole body, may burst later. Eyes swollen and water. Water from nose and mouth, cough (soft cough) and fast breathing, not eating, lame. Die in one week.**  **Pig bohonta- blisters. One village nearby has bohonta in cows.** |
| D | 2 | **Gaost. Hard hot lumps on body. May die. Happens some years here.** |
| E | 7 | **Goat: can’t walk properly, high temperature, only occurs sometimes, but trhen whole village affected, spreads rapidly, a few die. Treat with Dhunia and smoke- can recover.** |
| H | 3 | **Goat, mouth erosions, eye and nose discharge, lameness, skin lumps all over, shivers, dies.**  **Started last year. 200+ goats died – 225 goats belonging to 16 families, about 20 remained. ( Man 1: 15/16 goats died over 6 weeks, 1 infected; man 2: 15/16 dead; man3: 4/8 dead.)**  **“Kucheswan”** |
| I | 1 | **Lumps all over body, mouth and nose lesions, discharges, all affected goats die.**  **120+ households: > 1000 goats died (>90% of all goats dead)**  **Occurred after flood 2017- never seen this disease before.** |
| J | 2 | **Lumps all over body, erosions around nose and mouth, discharges, anorexia, die.**  **Occurred for the first time this year. All affected goats died. About 100 dead- 1/3^rd^ of village goat population.** |

**Kandh sangha**

| **Village / rank** | | **Description** |
| --- | --- | --- |
| F | 6 | **Working bullock**  **Can occur in cow too. Itchy.**  **Can’t work.**  **Parasites (lice?) – pruritus – maggots – apply petrol – chronic wound** |
| I | **5** | **Hump sore. Affects work** |

**Hafonee**

| **Village / rank** | | **Description** |
| --- | --- | --- |
| G | 4 | **Cow, occurs at any time of year. May die, takes time, a few animals affected. Saliva drips, can’t move, fever, not eating, occasional cough. Hard breathing? – not sure.** |
| I | **-** | **Fever, body shaking, can’t eat** |

**Geva- ghuti**

| **Village / rank** | | **Description** |
| --- | --- | --- |
| B | 9 | **Geva- ghuti (Dhena):** Tongue swells (cow), only one animal at a time, doesn’t spread between animals. Cow can’t eat and dies if untreated. Treatment- puncture tongue and put alum and dimorh (herb) in wound. |

**Bat hera**

| **Village / rank** | | **Description** |
| --- | --- | --- |
| J | 8 | **Teat blocked goat. One side of udder hard. Babies can’t feed. Goat not being milked, only feeding babies. (Occurs in 40%?)** |

**Poor growth goat**

| **Village / rank** | | **Description** |
| --- | --- | --- |
| G | 7 |  |

**Moh bis oni**

| **Village / rank** | | **Description** |
| --- | --- | --- |
| J | 5 | **Cow and calf leg swells then dies (BQ** |
| H | **-** | HS and BQ (said the letters), participants didn’t know local names. Used to cause deaths- hard breathing, now give vaccine- no problem. |

**How- phora**

| **Village / rank** | | **Description** |
| --- | --- | --- |
| J | 9 | **White liquid in cow dung- like white jelly. Animal is weak. No change to skin. Older cow is affected. Give preserved lemon- animal becomes well in 2 – 3 days.** |

**Anonymised Interview data**

**What is a ‘good’ animal?** How do you know?

Ox: size of thighs

Cow; length, belly size, udder (plump- good, flat no milk)

Look not important

**How do you know if an animal is healthy?** How do you know if an animal is unhealthy?

**What diseases/ problems are important for your animals?**

1. Fat belly: 1 y/o, very healthy, ate breakfast, stopped eating, went out to paddy. Found dead a few hours later, belly fat (distended) both sides.
2. Tongue disease: happens after flood every year Not same animals as hoof disease. Stop eating, salivate, ulcers on tongue. Not same animals as hoof disease.
3. Hoof/ foot disease: split appears along outside of hoof. Very lame. Become well again. Happens after flood every year. Sometimes few animals, sometimes all. Animal may/may not be infected again next year. Goats not affected
4. Swelling and crackling sound under skin (SC emphysema). Starts in one place then spreads rapidly. Stop eating, die in 12- 14hr. Skin not broken. Belly fat after a few hours.
5. Goat dysentery. After flood when fresh grass comes, every year, many affected (outbreak, whole village)die in 2-3 days, young and old affected.
6. Dysentery of duck and chicken. Dung white, die. All year (was just dry season)

**Can you teach me how you recognise the disease? Treat?** Prevent?

1. No name. No tx
2. No name. No Tx
3. Kura Phoata (‘hoof broken’). No Tx.
4. No name. Tx: Praying. Call Dr- slow to come, disease worse when arrived.
5. Hagoni (‘loose motion’). Tablets as per pharmacist (or “whoever on counter”) advice. Local tx: Hoo-ra leaf, Gurval leaf, jack fruit leaf. 90% die even with treatment.
6. Hagoni. No Tx.

**When did this last occur?**

1. Don’t know
2. After flood (2016)
3. After flood (2017)
4. January 2017
5. After flood. 4 goats died.
6. After flood. All household ducks and chickens died.

**What things limit how much your animals give?** ( provide for your household?)

Tiger last loss Dec 2016 cow killed tied up in paddy, seen by neighbour. Attack on cow in shed in 2000. Killed, tiger seen dragging away, then dropped.

Other wild animals.

**Young Animals**

Where do you keep your calves? Can we see please?

**How many of your cows/ buffs/ goats had a baby in the last year?**

5 cows, 4 gave birth

**Where are those baby animals now?**

1 dead flood

1 dead BQ

2 alive and at home

**When a calf is born, can you teach me about the first day of its life?**

(When should it first drink milk (colostrum)? How much? What can you do to help it to be healthy? Who helps it?)

Tie rice straw in mouth and round back of neck/ behind ears (then check suck) leave in place until breaks (approx. 30 minutes) or mother removes. Belief is it helps to drink.

Colostum (‘Phea- hom) when placenta falls (usually 1-2 hrs) milk off colostrum- 0.5 – 1 L varies with cow size. Remaining colostrum calf allowed to drink. Make colostrum sweets and eat.

No other treatment to calf.

Cow not milked again until calf 7 days old.

**How much milk does an older calf drink and how often does it get it?** (Can you teach me how you do this? Who is responsible for this?)

Milk in morning only, calf gets rest. Used to milk twice daily.

(for native cattle) Calf separated from cow overnight. Send calf to cow- starts let down- remove calf- milk cow empty– return calf – second let down – remove calf – milk again – calf gets rest of milk.

**Do your calves have any food apart from milk? Water?**( Where? How often? Who’s job?)

Calf drinks milk and eats grass from 1 month. Water bowl.

All family members do, whoever available.

**How much milk does your family get each day from your animals?** (What do you do with it? When do you first milk a cow/ buff /goat for drinking?)

Milk 1 – 2 L/ cow daily, dependant on cow size. Sufficient for family. Was selling 1L daily, now supply not enough to sell (since two dead calves).

**Adult Animals**

**Can you show me where your animals rest in the day? At night?**

Morning tie in paddy, evening feed and graze at paddy side, home in evening. Cooked food at dawn- rice husk, banana stem, salt, stay in shed overnight.

During flood, bring rice straw and cut grass.

Taken to river or water hole at paddy twice daily. Bowl of pump water at night.

**How do your animals get food? What**? (Who (family member) is responsible for this? Can we meet them please? How much land do you have? Can we see it?) **Where do your animals drink? How often?**

**Did you vaccinate your animals last year? Why / why not?**

No. Dr didn’t come.

Don’t know what to give. Don’t think about giving medicine to an animal which is not sick.

**Do you de-worm animals? How? Why/ why not?**

No. Sometimes see worms in dung- give sugar cane leaf.

Leaches attack navel in river- feed sugar cane leaf and mustard oil and camphor in wound (effective for leaches and flies)

**Do you do anything else to help your animals?**

**You live close to the forest reserve. Does this affect you?**

Elephant, rhino, buffalo, deer, boar destroy crops. Respondant spends every night in growing season in tungi, when crops ripe- many people in tungi. Use fire, shouting, torches, crackers from FD if they have them.

**Animal health education**

**Is there anyone who can treat your animals?** (What would you like the vet doctor to know more about?)

Vet doctor 5km, doesn’t usually come to village/ slow to attend, no one in village.

**Would you like to learn more about animal health? Why?**

Yes, save own cattle. Cow Rs 15 -17k, Ox Rs 22 – 25K

**What problems are there for people trying to learn about animal health in your village?** ( Why? Which ways of learning about animal health would be good for you (village meetings, classes, leaflets, radio broadcasts, internet information, etc)?)

No guidance when young, keen to learn. Wants 10 days training, then books- can someone provide this?

**What changes could make your farm to more productive/ better?**

Learning. Paddy is main enterprise so cattle get less attention.

**What do you think is the future of this farm? (**Who will you pass your knowledge of farming on to?)

Want to increase size of farm, buy cattle, buffalo, been planning for 8 years but paying for sons education and unable to afford both. If bigger farm, would not be able to labour for cash. Prefer farming, as getting older it is satisfying to grow farm.

Sons? If they get good jobs he will continue to look after farm. Dream is to buy land on high ground that won’t flood. Won’t have to take cattle to the road during the flood.

**Anything else you would like to tell us?**

Vet services would be a big morale booster. TCF vaccine programme protected animals, can we get this help for other problems. The medicine for this came from very far, we don’t have resources for that and I am very grateful to TCF for this.

Can 2 or 3 youths from our village be trained in basic veterinary care?

What about compensation policy from FD for crop damage? NP explained.

**2**

Village: Date: 16/3/17 Time: 12pm Interviewer: Andy Translator: Jadu

Location/ who else is present / what else is happening?

Home. Wife and mother present – asks them a lot, esp re illness

Name: Male/ ~~female:~~ Age: 49

**Your family and household**

**Who lives in your house?**

(Can we meet them please? What do they do? What do you want to do when grown up?)

Self

Wife

Son 23 learning to drive JCB

Son 20 Helping farm

**How is your family/ household supported?** (food, money, rent, etc).

Farming: paddy veg for house

Labour before, now stop to concentrate on farm

**What animals does your family/ household have? Why are your animals important?** (Can we see your animals please? Why do you keep them? How do animals help to support your household? )

Cow 2, 1 in milk

Calf 2, sell if money problem- usually happens (use like bank)

Goat 4 sell, don’t eat

Duck 2 eat eggs

Ox 2 draft

**What is a ‘good’ animal?** How do you know?

Ox: thigh muscle, body size, hump size

Cow body size, udder confirmation

Avoid unusual signs (features?)

Appearance not important but religion (superstition?) says black goat has more meat and better flavour

**How do you know if an animal is healthy?** How do you know if an animal is unhealthy?

Poor movement, not drinking water

**What diseases/ problems are important for your animals?**

1. “Kura Phata” Foot/hoof: lesion at side at coronary band and spreads around, occurs after flood, spreads rapidly between animals.
2. No name Tongue: saliva dripping, can’t eat, after flood, spreads rapidly between animals (considers separate problem to above). After flood, also dry season.
3. Loose teeth- Ox
4. “Dysentery” Goat dysentery: occurs after eating new grass after flood. Most die.
5. “Pahapori Dhora” Goat skin rash: bald, itchy. Dry season
6. “KuKuri” “Cona sagolay” Goat blindness: spreads, one eye or both, lacrimation, blephrospasm, eye turns white.

**Can you teach me how you recognise the disease? Treat?** Prevent?

1. Call Dr, usually won’t come, says can’t treat, must vaccinate. Buy tablets from pharmacy. Flies come, use Himax.
2. No Tx unless Dr comes
3. No Tx
4. Guava leaf, Hora leaf, Jack fruit leaf
5. Burn rice husk and apply ashes and haldi. Usually not effective.
6. Letiku (daal stem) tie around neck

**How often do these problems occur?**

1. 2014-2015
2. 2015
3. Can’t remember last time (i.e. long ago)
4. After flood 2016, every year
5. Every year- dry season
6. After flood 2016, can occur any time

**What things limit how much your animals give?** ( provide for your household?)

Nothing

Wife treats sick animals and milks cow

**Young Animals**

Where do you keep your calves? Can we see please?

**How many of your cows/ buffs/ goats had a baby in the last year?**

2 cows, 1 calf 1 alive and well,

Another calf 1.5 years old

**Where are those baby animals now?**

**When a calf is born, can you teach me about the first day of its life?**

(When should it first drink milk (colostrum)? How much? What can you do to help it to be healthy? Who helps it?)

Navel care- wash with mustard oil for 5-6 days and remove flies. Himax if flies come. Wash calf after birth (mustard oil and cloth), keep warm.

Colostrum: milk off 0.5 – 1L varies with cow size. Put calf on tit and leave to drink. Wife in charge.

Next milking 15 days

**How much milk does an older calf drink and how often does it get it?** (Can you teach me how you do this? Who is responsible for this?)

Calf full drinking for 15 days

Milk once daily, morning, all 4 teats, not empty

**Do your calves have any food apart from milk? Water?**( Where? How often? Who’s job?)

Grazing, drink on own (water?).

Cow tied in paddy, calf loose.

**How much milk does your family get each day from your animals?** (What do you do with it? When do you first milk a cow/ buff /goat for drinking?)

1. 1.5L / day. Sell 1L

**Adult Animals**

**Can you show me where your animals rest in the day? At night?**

Day: tied in paddy, night shed

**How do your animals get food? What**? (Who (family member) is responsible for this? Can we meet them please? How much land do you have? Can we see it?) **Where do your animals drink? How often?**

Salt water once daily

During flood: rice straw, cut grass, banana tree (whole tree, chopped ) w/ rice husk.

1 banana tree lasts 2 days. Tree grows in 6- 12 months, plant every year.

**Did you vaccinate your animals last year? Why / why not?**

Since 2015. Done by State Animal Husbandry Dept. Did not know about vaccination before that. Will continue.

**Do you de-worm animals? How? Why/ why not?**

No. Sometimes see worms in cow dung. Animal recovers.

**Do you do anything else to help your animals?**

**You live close to the forest reserve. Does this affect you?**

Crop raiding: Elephant, rhino. Elephants eat banana trees and sometimes injure people.

Tiger: killed cow in shed 3-4 years ago

**Animal health education**

**Is there anyone who can treat your animals?** (What would you like the vet doctor to know more about?)

No

**Would you like to learn more about animal health? Why?**

“I don’t have time, but my sons can learn”

**What problems are there for people trying to learn about animal health in your village?** ( Why? Which ways of learning about animal health would be good for you (village meetings, classes, leaflets, radio broadcasts, internet information, etc)?)

Sons should read books first, then go for practical training.

**What changes could make your farm to more productive/ better?**

“There is no money. If there was money I could consult the doctor more often.”

**What do you think is the future of this farm? (**Who will you pass your knowledge of farming on to?)

“I want to increase the number of animals as land for cultivation limited. If I buy in new breeds of cows then milk production could be increased.”

**Anything else you would like to tell us?**

“Protecting crops from tungi is hard work and I am up all night. The solar fence does not protect us from crop raiding. I have no cultivation land of my own. I work another man’s land and give him 50% of the crop. Crop raiding is a village problem.”

AH: “Is crop raiding more or less since the fence was put up?”

“Yes, crop raiding is less since the fence was built. The fence is helpful. Crop raiding is less this year. Our main problem is the flood. Now we are cropping in a different season, but this means we are increasing spending on water."

**3**

Village: Date: 17/3/17 Time: 9.30am Interviewer: Andy Translator: Jadu

Location/ who else is present / what else is happening?

Home, Mother present and very knowledgeable

Name: ~~Male~~/ female: Age: 30

**Your family and household**

**Who lives in your house?**

(Can we meet them please? What do they do? What do you want to do when grown up?)

Self

Younger brother : driver

Mother

Sister in law

Nephew: 3 yr

Uncle

Uncle wife

Son 15 years

Son 13 years

Daughter 10 years

**How is your family/ household supported?** (food, money, rent, etc).

Farming: paddy, mustard veg for house, occasional sale

Weaving and weaving trainer

Trading goods: store, farming goods. Own one car: transport goods and other driving

**What animals does your family/ household have? Why are your animals important?** (Can we see your animals please? Why do you keep them? How do animals help to support your household? )

Cow: 2, 1 in milk

Calf: 3 , may sell if males (or sell own Ox if old and train new). Use all calves.

Goat 12 - 13 sell, don’t eat (vegetarian)

Ox 2 draft

**What is a ‘good’ animal?** How do you know?

Cow: hair short and soft, navel slightly long- gives more milk

Ox: teeth

No black cows (“Black cows don’t suit this house”), only brown.

**How do you know if an animal is healthy?** How do you know if an animal is unhealthy?

Not eating (“main sign of illness”), Mood.

**What diseases/ problems are important for your animals?**

1. “Saboka” mouth Dz, saliva drips, tongue infected, lesions on mouth, lips and tongue, many animals, spreads between them. Occurs after flood.
2. “Kura-Phoata” broken hoof. Occurs after flood. Spreads rapidly to affect many animals. Saboka and kura-phoata may occur together.
3. “Dhoka- dingia” fat belly. Both sides distended, constipated, must pass dung to become well. Occurs after flood.
4. “Bhekulia” swollen neck. Occurs after flood, one animal at a time, die w/o Tx and must tx w/in 3d. Vacc controls.
5. “Letekua” eye disease (goats) 1 or both eyes, one animal at a time, any time of year. Eye turns white, later top layer comes away. Become well in 3 months.
6. Circling goat- no name. Aged goats. Go both ways, get very thin and won’t eat, head tilt, turn towards tilt. Always die. Cow/calf eat moria- guti, seeds get suck in teeth (intoxication?)
7. “Anthrax”Not grazing, fat belly, blood from nose, die in 3 hrs, sometimes 10mins. Animals (buffalos) died in forest last september, FD burned corpses, problem occurred after that.

**Can you teach me how you recognise the disease? Treat?** Prevent?

1. Salt water wash, KMnSO4 Sol if maggots
2. Tie in mud, use phenyl or ginger paste in shed to keep flies away
3. Black salt, sandur (paint for married lady head)in banana leaf- make parcel and swallow. Works in 1-2 hr. Preserved lemon by mouth.
4. House frog (dead) tied round neck (don’t know if this works)
5. Cow remove seeds from between teeth and wash teeth with lemon juice.

Call Dr for most problems- Kandhulimari (4km away)

**How often do these problems occur?**

1. After flood, all occurred this year (to Minalini’s animals?)
2. After flood
3. After flood
4. After flood
5. Any time
6. Any time
7. After flood

Less problems since started vaccinating.

**What things limit how much your animals give?** ( provide for your household?)

Tiger kills goats and cows. Took a goat from shed 4 years ago- saw pug marks.

Saw leopard in tree above shed 15 days ago.

**Young Animals**

Where do you keep your calves? Can we see please?

**How many of your cows/ buffs/ goats had a baby in the last year?**

2 cows, 2 calves 6-7 mths/o

**Where are those baby animals now?**

**When a calf is born, can you teach me about the first day of its life?**

(When should it first drink milk (colostrum)? How much? What can you do to help it to be healthy? Who helps it?)

Clean cow and calf.

Tie rice straw through mouth (10 -15 mins) and check movement of tongue – claf or mother break straw.

Cut tips off hooves- prevent big hoof problem,, goat same.

Colostrum (“Phae- Hoo”) : milk out 70%, leave rest for calf (approx. 1L house, 0.5L calf). Make sweets.

Next milking 7d, cow and calf together at all times until then.

**How much milk does an older calf drink and how often does it get it?** (Can you teach me how you do this? Who is responsible for this?)

Calf drinks for a few minutes- let down – remove calf- milk 2 teats empty- rest for calf.

When calf 3 m/o- milk twice daily

**Do your calves have any food apart from milk? Water?**( Where? How often? Who’s job?)

From 3 months grazing and cooked food (veg, banana tree, rice husk)

“Sicora” tick problems and leaches: “How do we get rid of these? I realise ticks are important for the cycle of life, they provide food for the birds and small creatures, but they feed on our animals. I use medicine that I pour along the calf’s backbone, but it makes the calf weak. Is there another way to deal with them?”

**How much milk does your family get each day from your animals?** (What do you do with it? When do you first milk a cow/ buff /goat for drinking?)

Once daily milking 2.5 L/cow Twice daily 3.5 L/cow Use 1L daily, sell surplus.

Jersey cross cows, once had a cow that gave 5L morning and 2L evening

**Adult Animals**

**Can you show me where your animals rest in the day? At night?**

Shed at night, tied in paddy in day- no shade in paddy, if too hot take to shade of tree.

**How do your animals get food? What**? (Who (family member) is responsible for this? Can we meet them please? How much land do you have? Can we see it?) **Where do your animals drink? How often?**

Grazing, cooked food at night.

Water taken to field at noon, very hot day- take 2-3 times. Rice water/ rice washing water w/ salt added. All family members do.

**Did you vaccinate your animals last year? Why / why not?**

Yes. 2015, 2016. Prevents Dz. Occurs d/t Forest Dept initiatives to protect wild animals. Consultation of Forest Dept and Eco Development Committee in order to take advantage of Dz prevention initiatives. I have seen animals being vaccinated at times for my whole life.

**Do you de-worm animals? How? Why/ why not?**

Yes. Dr medicine or pharmacy shop

“Mackie pilou” (maggots) wash wound and apply petrol, kerosene or turpentine. Use Himax on wounds to prevent.

“Ouja”- faith healer (not a priest)- pray and perform a ritual then give something in a wrap of banana leaves which the animal must eat. Treats maggots. Is it effective?- not sure.

**Do you do anything else to help your animals?**

Use smoke to prevent mosquitos in shed or mosquito nets

Nose rope placed at 5 – 7 years. Make animal lay down (cast) tie all legs, piece nose w/ bamboo spike and then use it to draw rope through.

**You live close to the forest reserve. Does this affect you?**

Spread of Dz from wild animals.

Crop raiding- elephant “hati”(biggest problem), also wild boar “guar-ri”, deer “mus-tad”, wild buffalo “mo-ho”. Rhino “gore” less often.

**Animal health education**

**Is there anyone who can treat your animals?** (What would you like the vet doctor to know more about?)

Vet Dr 5km away. “Not very expert. One buffalo died from wtong treatment a few years back. Buffalo had Dz, I told the Dr and he vaccinationed the buffalo anyway and it died. This was years ago”.

**Would you like to learn more about animal health? Why?**

Yes. “ I didn’t learn when I was young as my father and brother were alive and they did those things. Now I need to know more as I am running the house.”

**What problems are there for people trying to learn about animal health in your village?** ( Why? Which ways of learning about animal health would be good for you (village meetings, classes, leaflets, radio broadcasts, internet information, etc)?)

I have some time available. “Learning theory from books is good, but I need practical training so that I can really learn to do these things.”

**What changes could make your farm to more productive/ better?**

I need help or money before I can make changes. I would like to buy more cows but then I would have to hire more men to help look after them. I did a training course, Dairy Development Trainin.

**What do you think is the future of this farm? (**Who will you pass your knowledge of farming on to?)

I want to expand

I don’t know (who I will pass my knowledge on to).

**Anything else you would like to tell us?**

“It would be good if you (Andy) did some training in the days while you are here. It would be very helpful ti us (the villagers)”

**4**

Village: Date: 17/3/17 Time: 11.30am Interviewer: Andy Translator: Jadu

Location/ who else is present / what else is happening?

Home. Mother present and very knowledgeable

Name: Papul Bora Male/ ~~female~~: Age: 27

**Your family and household**

**Who lives in your house?**

(Can we meet them please? What do they do? What do you want to do when grown up?)

Self: Driver also

Father: 50

Mother: 45 All working farm

Sister: 17

Brother: 21

**How is your family/ household supported?** (food, money, rent, etc).

Farming: paddy, mustard, potato veg for house, occasional sale

Driving

**What animals does your family/ household have? Why are your animals important?** (Can we see your animals please? Why do you keep them? How do animals help to support your household? )

Cow: 3, milk and dung for cultivation

Calf: 2, sell if males at 8 – 10 mth/o, keep females.

Goat 4, sell, eat at festivals occasionally

Ox 0. Sold- old and sick (15 yr/o). Sold in market. Currently rent tractor (cultivation machine) when required, costs Rs300 / bihar (unit of agricultural land). Intend to buy more Ox before flood so don’t have to rent tractor.

Chicken 10 – 15. Sell, Also eat.

**What is a ‘good’ animal?** How do you know?

Ox: Legs, teeth and hooves.

Cow: Feel sometning inside when looking at cow. Also, is it giving a lot of milk? What is the cow eating? Udder and teat size is also important.

“Colour should suit the house. Black and red for this house.” Colour is not main consideration, other things come first.

**How do you know if an animal is healthy?** How do you know if an animal is unhealthy?

“Not eating. Laying down- assume something is wrong. A healthy cow is always active. Eats a drinks a lot.”

**What diseases/ problems are important for your animals?**

1. “Kura-Phata” broken hoof. Lame, mostly one leg. Doesn’t want to walk. Wounds start. Saliva drips from mouth, will eat. Sometimes one, sometimes all. All time of year, usually starts in season when mud on paddy (June- July).
2. “Liquid from eye- no name” eye disease (goats) 1 or both eyes, usually one animal at a time, not blind but can’t see. First looks. Become well, sometimes need Dr medicine, otherwise just wash eye with water.
3. Wound on neck for working ox. Dr medicine. Wash wounds and apply powder for flies.
4. Fat Belly “Pet Fulla”- “Pet- Bikh”(belly pain). Won’t eat, lays around, may die. Get Dr- gives injection or tablets. No local treatment. Any animal, any time of year.
5. Dysentery after flood when new grass grows. Feed rice straw as treatment. May get well, usually don’t die. Any goat, any time.

**Can you teach me how you recognise the disease? Treat?** Prevent?

**How often do these problems occur?**

1. 2015
2. 2016
3. 2012
4. Never happened to these cattle
5. 1 cow 2016

**What things limit how much your animals give?** ( provide for your household?)

“Everything is 100%, because everything will occur. We have no problems except Dz.”

**Young Animals**

Where do you keep your calves? Can we see please?

**How many of your cows/ buffs/ goats had a baby in the last year?**

3 cows, 3 calves

**Where are those baby animals now?**

1 died after flood. Not eating after flood finished.

**When a calf is born, can you teach me about the first day of its life?**

(When should it first drink milk (colostrum)? How much? What can you do to help it to be healthy? Who helps it?)

“Phoa- Ho”- milk cow out. Make sweets. Remove 0.5 – 1 L. Yellow colour faded in milk before calf drinks.

Clean calf.

Tie rice straw through mouth (30 mins) – makes calf strong at suckling.

Start fire to warm calf if born in winter.

Next milking 5-6d, cow and calf together at all times until then.

**How much milk does an older calf drink and how often does it get it?** (Can you teach me how you do this? Who is responsible for this?)

Tie calf separate from cow until midnight- loose in morning wait 30 mins, then milk cow. Calf drinks first.

All family members do.

Milk once daily for 15 days, then twice daily

**Do your calves have any food apart from milk? Water?**( Where? How often? Who’s job?)

When starts grazing give cooked food (rice polis and rice husk). Banana tree. In evening, father does.

**How much milk does your family get each day from your animals?** (What do you do with it? When do you first milk a cow/ buff /goat for drinking?)

1. 2L / cow / day. Twice daily = 4L.

**Adult Animals**

**Can you show me where your animals rest in the day? At night?**

Free graze, no tie, shed at night

**How do your animals get food? What**? (Who (family member) is responsible for this? Can we meet them please? How much land do you have? Can we see it?) **Where do your animals drink? How often?**

Grazing, cooked food at night- rice polis and banana tree.

Store fodder for flood time.

Self- drinking at river. Don’t give water at night but water in cooked food.

**Did you vaccinate your animals last year? Why / why not?**

Yes. 2016 first time. Other people told me that it will reduce infections. All kinds of disease.

**Do you de-worm animals? How? Why/ why not?**

No. I don’t see worms.

**Do you do anything else to help your animals?**

Nose rope placed at 5 years. Father does. He lays ox down (cast) and tie all legs, piece nose w/ bamboo spike and then use it to draw rope through.

**You live close to the forest reserve. Does this affect you?**

Elephant and boar destroy crops.

**Animal health education**

**Is there anyone who can treat your animals?** (What would you like the vet doctor to know more about?)

There is a village man with some knowledge, he had Govt training.

**Would you like to learn more about animal health? Why?**

Yes. “The I can help family cattle and neighbours.”

**What problems are there for people trying to learn about animal health in your village?** ( Why? Which ways of learning about animal health would be good for you (village meetings, classes, leaflets, radio broadcasts, internet information, etc)?)

I lack scope of learning (school/ institute) money is also an issue.

Practical learning is best. Meetings are ok. Leaflets are ok, but not my preferred way. Radio can only be a help after practical teaching.

**What changes could make your farm to more productive/ better?**

More cows and feed well for better milk production

**What do you think is the future of this farm? (**Who will you pass your knowledge of farming on to?)

I want to learn about vet care and buy more cattle, ones with better milk production that are easy to handle.

I want to carry on here. I think about it a lot.

**Anything else you would like to tell us?**

“If a few people are trained (as gau sewek) it will be very good for the village. We don’t have to wait for veterinary doctor and can look after cattle in a very good way.”

**5**

Village: Date: 17/3/17 Time: 1.30pm Interviewer: Andy Translator: Jadu

Location/ who else is present / what else is happening?

Home, Mother, wife, children. Asks wife a lot.

Name: Male/ ~~female~~: Age: 39

**Your family and household**

**Who lives in your house?**

(Can we meet them please? What do they do? What do you want to do when grown up?)

Self: teacher also (village school)

Wife

Mother:

Son: 13

Daughter: 7 Have own will, I would like them to get good jobs. He likes electronics, she likes dance and music.

**How is your family/ household supported?** (food, money, rent, etc).

Farming: paddy, mustard, veg for house and sale

Teaching (school not fully supported by Govt)

Small shop at house, sell veg and a few other items

**What animals does your family/ household have? Why are your animals important?** (Can we see your animals please? Why do you keep them? How do animals help to support your household? )

Cow: 2, milk and dung for cultivation

Calf: 3, sell when need money (use as a bank) or keep to increase herd. One is older (3yrs) calf.

Goat 4, sell only, vegetarian.

Ox 2. cultivation

**What is a ‘good’ animal?** How do you know?

Ox: Teeth (age), physical figure. Touch body and feel power.

Cow: Body size and heavy backside. And big udder.

For this house, red and white. Not main thing.

**How do you know if an animal is healthy?** How do you know if an animal is unhealthy?

Healthy: horns and legs

Unhealthy: Mood. Laying down. Not eating. Movement

**What diseases/ problems are important for your animals?**

1. “Saboka”: foot wound, salivation, lesion in mouth, Many animals, spreads rapidly.
2. “Kura-Phata” Wounds round foot. Saliva. Sores in mouth. Many animals. These are the same disease (some discussion occurred).occurs after flood. May die w/o Tx, doesn’t cause abortion. Same cow not affected every year.
3. “Gro- honi” dysentery, goat and cow, occurs from eating the first grass after the flood.
4. “Pet Fulla”- fat belly. Decrease RR, breathlessness. Dies w/o Tx in 1 - 1.5 hours. Cow, goat and calf. One animal at a time. Occurs at any time. Occurs in shed and at pasture.

**Can you teach me how you recognise the disease? Treat?** Prevent?

1. KMnSO4 and water wash feet, mouth also. Rarely die. If Mackie pulou come- powder camphor and apply (possibly pack wounds) very effective.
2. As above
3. Dr Tx. Also local leves (animal eats) can’t name). Preserved lemon.
4. Black salt in water- effective in 30 mins- hear gas release.

**How often do these problems occur?**

1. After flood 2016 (neighbours animals) been 5 years since happened to own animals.
2. As above
3. After flood 2016
4. Last case 7 years ago (to own animals.

**What things limit how much your animals give?** ( provide for your household?)

Flood

Sufficient fodder not always available

Flies and mosquitos

**Young Animals**

Where do you keep your calves? Can we see please?

**How many of your cows/ buffs/ goats had a baby in the last year?**

2 cows, 2 calves this year, plus one older.

**Where are those baby animals now?**

Here.

**When a calf is born, can you teach me about the first day of its life?**

(When should it first drink milk (colostrum)? How much? What can you do to help it to be healthy? Who helps it?)

Clean calf. Make fire to warm calf in winter. Help to stand.

Milk all 1 litre “yellow milk”“Phaa- Ho”- milking all 4 teats. Take for house. Make sweets. Rest of phaa ho for calf. This is 30- 40% remaining for calf (guess?).

Next milking 5-6d, cow and calf together at all times until then.

**How much milk does an older calf drink and how often does it get it?** (Can you teach me how you do this? Who is responsible for this?)

Milk once daily. Get 1 – 1.5L from milking all 4 teats.

Calf separate from cow overnight. Release calf, after calf drinking for a few moments- remove calf and milk cow

Husband teaching in morning, so other family members responsible.

Cow and calf together in day

**Do your calves have any food apart from milk? Water?**( Where? How often? Who’s job?)

Calf starts grazing after 1 month. Given rice water also. Drinks water with cow.

Everyone’s responsibility.

**How much milk does your family get each day from your animals?** (What do you do with it? When do you first milk a cow/ buff /goat for drinking?)

- 1. L / cow / day. Therefore 3 L daily. Drink 1L, sell 2L

**Adult Animals**

**Can you show me where your animals rest in the day? At night?**

Day- tied in paddy to graze, shed at night. No shade in paddy, if very hot we take to shade.

**How do your animals get food? What**? (Who (family member) is responsible for this? Can we meet them please? How much land do you have? Can we see it?) **Where do your animals drink? How often?**

Grazing paddy.

Cooked food given at night- rice husk and vegetables.

During flood time feed banana tree also.

Water given morning and night, during summer at midday also.

**Did you vaccinate your animals last year? Why / why not?**

Yes. Last 10 years. We o this after the flood.

**Do you de-worm animals? How? Why/ why not?**

De-worming tablets given to calves at 6 – 12 months.

**Do you do anything else to help your animals?**

**You live close to the forest reserve. Does this affect you?**

Crop raiding by elephant and wild boar.

Predation by tigers, though this has never happened to my animals.

**Animal health education**

**Is there anyone who can treat your animals?** (What would you like the vet doctor to know more about?)

No.

**Would you like to learn more about animal health? Why?**

Yes. “I can do this for my own cattle and my community.”

**What problems are there for people trying to learn about animal health in your village?** ( Why? Which ways of learning about animal health would be good for you (village meetings, classes, leaflets, radio broadcasts, internet information, etc)?)

I have had a lack of exposure to this type of learning. I can learn part time but the training must be in a local place. Short training is better for me.

**What changes could make your farm to more productive/ better?**

I want to introduce hybrid animals with better genetics to increase milk production

**What do you think is the future of this farm? (**Who will you pass your knowledge of farming on to?)

We are going along but we are too busy to expand.

My son will probably continue to farm here.

**Anything else you would like to tell us?**

**Interviews 2017**

**6**

**Village**: **Date**: 17/3/17 **Time**: 3pm

Interviewer: Andy Translator: Jadu

**Location/ who else is present / what else is happening?**

Outside house being built. Mother present, children and other family members coming and going.

**Name**: (and mother) **Male/ ~~female:~~** **Age:** 49

**Your family and household**

**Who lives in your house?**

(Can we meet them please? What do they do? What do you want to do when grown up?)

Self

Mother

Sister

Brother (works for defence service)

Brother wife

Twin daughter: 11 “I want my children to be self- independent and become educated.”

Twin daughter: 11 “The future depends on them.”

Son: 6

Brother daughter: 2

Wife present but not listed by interviewee.

**How is your family/ household supported?** (food, money, rent, etc).

Farming: Paddy, mustard, Peas, Daal. Vegetables

Brother’s salary

Dairy farm (“jerseys”- local expression which appears to mean dairy cattle) at another location.

**What animals does your family/ household have? Why are your animals important?** (Can we see your animals please? Why do you keep them? How do animals help to support your household? )

Cow: 4 milk, calves, dung (biogas made for cooking)

Calf: 0 (There were 2, but they are dead). Keep females to increase cow numbers, sell males

Goat: 13 Sell to butcher. Occasionally family eat one goat if there is a wedding or other event.

Ox: 0

Hire tractor for cultivation. It is difficult to raise ox. One day we will probably buy a tractor

**What is a ‘good’ animal?** How do you know?

Cow: milk veins, facial structure, especially eyes. Udder size.

**How do you know if an animal is healthy?** How do you know if an animal is unhealthy?

Eating style. Style of motion, coat condition

**What diseases/ problems are important for your animals?**

1. “Pelu” (worms)(goat): ‘One goat mother died one month after giving birth, worms were the cause. No I did not see the worms, but that is what I suspect (para-phrased)
2. “Muri- goroni”: (circling goat) Head tilt, turns towards head tilt. Aged (adult?) goat only. One goat at a time. Always die.
3. “Jor” or “Zor” (fever); goat or cow. Know by hot ears.
4. “Pet- Fulla” (fat belly): occurs after flood, gas problem, all animals may be affected. Occurs in paddy or shed. Die unless treated.
5. “Hagoni” (dysentery): After flood, animals weaken and may die if untreated.
6. “Soboka”: During flood. Foot problems occur first, if cow licks wound tongue problem may occur also with salivation. Get well if treated, otherwise may die. Same animal may or may not get the infection two years in a row. Very occasionally abortion happens in pregnant animals. Does not occur in goats.

**Can you teach me how you recognise the disease? Treat?** Prevent?

1. Worm tablets
2. Few recover- die in 1 month
3. Paracetamol, may die if untreated, will become well with Dr injections
4. Dr Tx or prescription from Dr.
5. “Zorba” leaf, Guava leaf, make paste, wrap in banana leaf and give by mouth. Very effective.

**How often do these problems occur?**

1. 2 years ago (dead goat detailed above)
2. January 2017
3. 5 years ago (“jersey”)
4. 5 years ago (same animal as above)
5. Has not occurred in my animals
6. 2014- 2015

**What things limit how much your animals give?** (provide for your household?)

“Dhar” (specific type of fly): bite cows and causes blood loss (only “jerseys”)

**Young Animals**

Where do you keep your calves? Can we see please?

**How many of your cows/ buffs/ goats had a baby in the last year?**

4 cows, 2 calved

**Where are those baby animals now?**

Dead. 1 sudden death at 6 months, 1 died of 15 days from “weakness”

**When a calf is born, can you teach me about the first day of its life?**

(When should it first drink milk (colostrum)? How much? What can you do to help it to be healthy? Who helps it?)

Clean calf, clean udder, put calf to suck. 1^st^ milk (“Phaa- Ho) calf drinks. Drinks 1 -1 .5L (how do they know this?).Then milk out.

Mustard oil in calf mouth (one tiny cup).

Next milking- next day.

**How much milk does an older calf drink and how often does it get it?** (Can you teach me how you do this? Who is responsible for this?)

In morning calf drinks then we milk the cow. Then the calf roams free for the day and goes back. At evening the calf drinks again. Then cow milked again in the evening.

“If you don’t milk a cow empty then the milk clots in the cow’s udder. If this happens the cow must have an injection (antibiotic?) to prevent illness and the udder milked empty.”

**Do your calves have any food apart from milk? Water?** (Where? How often? Who’s job?)

Grass. Cooked food eaten from one month old: Rice polis, rice husk, vegetables, morning and evening.

Water twice daily.

**How much milk does your family get each day from your animals?** (What do you do with it? When do you first milk a cow/ buff /goat for drinking?)

12 L /day / cow

2 cows in milk, 2 cows pregnant not in milk.

**Adult Animals**

**Can you show me where your animals rest in the day? At night?**

In shed at all times

**How do your animals get food? What**? (Who (family member) is responsible for this? Can we meet them please? How much land do you have? Can we see it?) **Where do your animals drink? How often?**

Rice straw, green folder, rice husk, wheat husk, mineral powder and calcium.

Cooked food w/ water

100L water / day /cow

**Did you vaccinate your animals last year? Why / why not?**

Yes, since I bought in Jerseys. 10 years.

No Soboka in my cows.

**Do you de-worm animals? How? Why/ why not?**

Yes. Goats once per year

Cows. 4 times per year

Tablets from pharmacy.

**Do you do anything else to help your animals?**

Wash daily

Spray for insects on cows

**You live close to the forest reserve. Does this affect you?**

Spread of disease from wild animals to domestic animals.

Everything else is positive for kaziranga.

**Animal health education**

**Is there anyone who can treat your animals?** (What would you like the vet doctor to know more about?)

No. Dr/ compounder in Sabzuri 5km.

**Would you like to learn more about animal health? Why?**

Yes. Improve productivity.

**What problems are there for people trying to learn about animal health in your village?** ( Why? Which ways of learning about animal health would be good for you (village meetings, classes, leaflets, radio broadcasts, internet information, etc)?)

Lack of educational environment

Education teaching in farms with face to face teaching.

**What changes could make your farm to more productive/ better?**

Need more green grass. I need equipment (machinery) to chop forage.

**What do you think is the future of this farm? (**Who will you pass your knowledge of farming on to?)

I want to increase the farm size with increased number of cows and increased cow size.

**Anything else you would like to tell us?**

“Welcome to our village. I hope that through education programme all the people of the village can gain good animal health knowledge.”

**7**

**Village**: **Date**: 18/3/17 **Time**: 9.30am

Interviewer: Andy Translator: Jadu

**Location/ who else is present / what else is happening?**

Home

**Name**: Farmer and mother **Male/ female:** **Age:** 42 and 65

**Your family and household**

**Who lives in your house?**

(Can we meet them please? What do they do? What do you want to do when grown up?)

Self:

Father

Mother

Wife: 40

Brother: 38

Sister in law: 35

Son: 16

Niece: 5

Grandson: 11 mths

**How is your family/ household supported?** (food, money, rent, etc).

Farming: paddy, mustard, veg for house

Daily labour (self, brother, sister in law.

**What animals does your family/ household have? Why are your animals important?** (Can we see your animals please? Why do you keep them? How do animals help to support your household? )

Cow: 1 (just bought)

Calf

Goat: 4

Duck

Chicken: 6

Ox: 2

**What is a ‘good’ animal?** How do you know?

(Cow and Ox): Soft hair, strong legs, long tail, hoof “tight shape, pointy, not spread”

Cow: Backside- strong, udder size, check history of cows mother- how much milk.

**How do you know if an animal is healthy?** How do you know if an animal is unhealthy?

Good mood, physical condition = healthy

Laying down, not cudding = unhealthy

**What diseases/ problems are important for your animals?**

1)”Pet phulla” (fat belly): one side- cow gets tired, then gets breathing problem. Occurs in shed and paddy, mostly in winter, sudden onset, usually survive, sometimes die even with Tx. Call Dr if local medicine not effective, Dr medicine usually effective.

2) “Soboka” or “Kura- phatta”: can’t walk, wound runs around top of hoof, blood comes, then flies and maggots. Sometimes don’t eat and saliva drips. Infection may also occur in mouth and nose. Occurs after flood, spreads fast and can affect every animal. If an animal catches one year, it usually doesn’t catch next year.

3)”Gol- phulla” also called “Bhekulia”: Goats also affected. Swelling of neck (chin, running down ventral throat), weaker day by day, stop eating. After 12- 13 days- become well again.

4)”Basanta ulua” (skin pox): cow- whole body, hair falls, skin grey, skin falls, normal skin below. Itchy, animal scratches. Don’t eat, often die. Flys bother the cow and maggots come.

5) ”Phapora dhara” (skin rise in goats): occurs in summer season, spreads from goat to goat, dirty sheds- goat lies in faeces. Scratches- dandruff falls- very itchy- hair falls, skin becomes white and very thick.

6) “Letekua” (eye problem/ blindness): goats. One or both eyes, spreads from goat to goat. Occurs at any time, water from eye- water is clear or white, goat shuts eye or blinks a lot. Then eye goes white. With local treatment animal does not go blind.

**Can you teach me how you recognise the disease? Treat?** Prevent?

1. Black salt and cindhur (married women’s tikka paint). Or mud from rat hole, mix with water, spread on body (religious belief) and jump over cow one or two times.
2. Tie cattle in mud to cover wounds on hooves. Use Dr Medicine against flies from pharmacy. Separate animals with Soboka.
3. Tie frog (common house frog) around neck- tie around neck alive. Leave frog there until frog dies or animal well.
4. Maggots- wash wounds before dawn, don’t put anything inside wound. Use mosquito nets or smoke to keep flies away. Dr medicine for maggots also.
5. Tobacco with water and mustard oil- rub body- daily for 3 days. Or Dr injection, may give up to 3 injections.
6. Stick from Letekua tree- when stick becomes dry animal recovers.

**How often do these problems occur?**

1. 10 – 11yrs ago in this home
2. Not at this house for 4 – 5 yrs.
3. 7 years
4. Never, but I am concerned about this disease
5. 2 years
6. 4years

**What things limit how much your animals give?** ( provide for your household?)

**Young Animals**

Where do you keep your calves? Can we see please?

**How many of your cows/ buffs/ goats had a baby in the last year?**

0

**Where are those baby animals now?**

**When a calf is born, can you teach me about the first day of its life?**

(When should it first drink milk (colostrum)? How much? What can you do to help it to be healthy? Who helps it?)

Daytime- cow licks calf clean

Night time- make a fire to warm calf and boil water

Wash udder w/ warm water

Get calf to drink ASAP. If calf stumbles when it starts to walk, steady it and take to teat. Can use fingers to encourage to suck and open mouth if required.

Take no “Pha-Ho”.

Give cow warm water to drink and placenta falls away quickly. (What do you do if the placenta doesn’t fall away quickly?) The placenta always falls.

Keep the navel clean to prevent flies.

**How much milk does an older calf drink and how often does it get it?** (Can you teach me how you do this? Who is responsible for this?)

[Mother takes over]

Take a little milk at 3 days, properly milk at 7 d- morning only.

Milk 4 teats and take 75% of milk.

After 15 – 30 days milk twice daily.

**Do your calves have any food apart from milk? Water?**( Where? How often? Who’s job?)

Rice water after 15 days. Grazing.

Shares cows feed: cooked food: rice polis, veg, banana stems and banana tree.

Every ones job, but mainly done by older people as young people working at labour.

**How much milk does your family get each day from your animals?** (What do you do with it? When do you first milk a cow/ buff /goat for drinking?)

Once daily milking- 2L daily

Twice daily milking 2.5L daily

**Adult Animals**

**Can you show me where your animals rest in the day? At night?**

Day- paddy, night- shed

**How do your animals get food? What**? (Who (family member) is responsible for this? Can we meet them please? How much land do you have? Can we see it?) **Where do your animals drink? How often?**

Rice straw- quantity given varies with availability- always give during flood, give at other times if plenty.

Cooked food in evening all year round

Salt water noon and evening.

Dhania stems also used as fodder.

**Did you vaccinate your animals last year? Why / why not?**

Yes. Cows and Ox for last 10 years. Protects from Soboka and other diseases.

Use because Forest Department initiatives provide for free.

**Do you de-worm animals? How? Why/ why not?**

If the Foresrt Department or some NGO provide, then we use. Tablets and injections for cows and ox. Sometimes I see small white (flat?) worms in dung sometimes. There is no local medicine for this.

**Do you do anything else to help your animals?**

**You live close to the forest reserve. Does this affect you?**

Crop raiding by elephant and boar, not usually rhino.

**Animal health education**

**Is there anyone who can treat your animals?** (What would you like the vet doctor to know more about?)

Yes. Kanholi Murray 2km and Sabzuri 5 km.

Dr is very good and knows a lot but I have to pay.

**Would you like to learn more about animal health? Why?**

Yes. I will treat my own animals and advise villagers.

**What problems are there for people trying to learn about animal health in your village?** ( Why? Which ways of learning about animal health would be good for you (village meetings, classes, leaflets, radio broadcasts, internet information, etc)?)

Lack of resources.

I am an older lady and may struggle to learn but the young people may struggle for a lack of time.

People are poor and go for daily labour so they lack the time to read.

Training with a good teacher. If a training course was provided it would be up to the individual to make the time or not.

**What changes could make your farm to more productive/ better?**

?

**What do you think is the future of this farm? (**Who will you pass your knowledge of farming on to?)

Sons and grandsons

**Anything else you would like to tell us?**

**8**

**Village**: **Date**: 18 /3/17 **Time**: 11am

Interviewer: Andy Translator: Jadu

**Location/ who else is present / what else is happening?**

Middle street, home

**Name**: **~~Male~~/ female:** **Age:** 25

**Your family and household**

**Who lives in your house?**

(Can we meet them please? What do they do? What do you want to do when grown up?)

Self: Primary school teacher

Husband: 32, business, small grocery shop at house

**How is your family/ household supported?** (food, money, rent, etc).

Business

Farming: paddy, mustard

Teacher

**What animals does your family/ household have? Why are your animals important?** (Can we see your animals please? Why do you keep them? How do animals help to support your household? )

Cow: 2 sell calves, milk for house

Calf: 1

Goat: 4 sell

Duck

Chicken: 15 sell, eat, eggs, more chickens

Ox

Rent tractor when required

**What is a ‘good’ animal?** How do you know?

Good condition, eating well, good walking style. If these are poor then cow is weak or unhealthy

Husband buys cows, may go with friend

**How do you know if an animal is healthy?** How do you know if an animal is unhealthy?

**What diseases/ problems are important for your animals?**

1) ”Pet phulla”: fat belly- any time of year, 1 or 2 animals, in paddy or shed, sometimes whole belly swells, sometimes one side. Stop E/D/U/F. Become well, never die.

2) “Kura- phatta”: every animal, after flood. Stumble to walk, sometimes blood from foot, but always starts at foot. Eating less. Maggots go between hooves. Sometimes tongue disease too with saliva dropping but few like this compared with numbers with feet.

3) “Phapora Dhora” (Skin rash goat): any time of year, starts on one goat and spreads between animals. Starts on one area of goat and spreads. Whole body dry, hair falls in places, skin below is white in colour, thickened, dry flaky with many deep wrinkles.

4) “Dysentery” (goat dysentery): summer, after flood, especially when fresh grass- 50% of goats.

**Can you teach me how you recognise the disease? Treat?** Prevent?

1. Lemon juice, if not effective Dr injection (or tablets if injection not available.
2. KMnSO4 soln spray, fly spray, himax. Maggots- pull out of wound with a stick, apply topicure or himax.
3. Dr medicine- injection given one or more times
4. Dr injection- effective

**How often do these problems occur?**

1. After flood 2016
2. After flood 2015
3. Summer 2016
4. 10 days ago, became well.

**What things limit how much your animals give?** ( provide for your household?)

Tiger kills goats and cows. Calf killed last year. 2 goats also killed last year (in two months). These killings occurred during day time, near the river (at park boundary, used for grazing and watering animals), other villagers saw. This occurred over a period of 6 months.

**Young Animals**

Where do you keep your calves? Can we see please?

**How many of your cows/ buffs/ goats had a baby in the last year?**

2 cows – 2 calves

**Where are those baby animals now?**

1 living here

1 eaten by tiger

**When a calf is born, can you teach me about the first day of its life?**

(When should it first drink milk (colostrum)? How much? What can you do to help it to be healthy? Who helps it?)

Give cow cooked food- rice polis, veg, banana tree.

“Phay Who”(yellow milk)- take approx. 0.5L depend on cow size, drawn from all 4 teats

Cook phay- who (boil w/ sugar for 10- 15 mins until it boils down to make sweets). Give some sweets to cow and calf.

Calf drinks after milking is finished- approximately 1 – 1.5 hours old if he stands on own. Assist the calf to stand and suckle if required.

Nothing else is done.

First 7 – 10 days cow and calf together at all times.

**How much milk does an older calf drink and how often does it get it?** (Can you teach me how you do this? Who is responsible for this?)

Start milking cow at 7 – 10 days. Morning milking. Cow and calf separate at night- in the morning the calf drinks a little- then milk all 4 teats. Not milked empty- take 1 L / day.

Husband does.

**Do your calves have any food apart from milk? Water?**( Where? How often? Who’s job?)

No food until teeth grow (3 – 6 months).

Cooked meal- rice polis, veg, banana- same as mother

Water noon and evening.

Husband and wife do.

**How much milk does your family get each day from your animals?** (What do you do with it? When do you first milk a cow/ buff /goat for drinking?)

1 L / cow / day. Sell little only.

**Adult Animals**

**Can you show me where your animals rest in the day? At night?**

Shed night, paddy day

**How do your animals get food? What**? (Who (family member) is responsible for this? Can we meet them please? How much land do you have? Can we see it?) **Where do your animals drink? How often?**

Water at noon and evening

Cooked food in the evening

Husband does.

**Did you vaccinate your animals last year? Why / why not?**

Yes. Been doing for 7 years. Prevents Dz such as skin rash

**Do you de-worm animals? How? Why/ why not?**

Yes. Every 3 months. Injection or tablet. Never see worms in dung

**Do you do anything else to help your animals?**

**You live close to the forest reserve. Does this affect you?**

Crop raiding by elephants and wild boar. Only occasionally rhino.

Monkeys (Rhesus macaques) invade food store

**Animal health education**

**Is there anyone who can treat your animals?** (What would you like the vet doctor to know more about?)

Dr -5km (Sabjuri)

**Would you like to learn more about animal health? Why?**

Yes. Dr is not always available. I would like to learn more from doctors.

**What problems are there for people trying to learn about animal health in your village?** ( Why? Which ways of learning about animal health would be good for you (village meetings, classes, leaflets, radio broadcasts, internet information, etc)?)

“Training selected people would be more helpful. We lack the proper resources for learning here.”

“Teaching from someone with experience about animal health and farming would be the best way for people to learn and apply knowledge.”

(Reading materials?)” Yes, people can learn from them. A few might be interested, but not all. People here are too busy cultivating food.”

“The literacy rate here is about 30% (for what language? Assamese?). Yes, Assamese. A few people here can read Hindi, and English too. The young people read better, the old people less.”

**What changes could make your farm to more productive/ better?**

Don’t know

**What do you think is the future of this farm? (**Who will you pass your knowledge of farming on to?)

I would like mb son to have a good job

**Anything else you would like to tell us?**

“It is very good that you have come to us. This is a very good initiative. No one has ever come here before to do any kind of veterinary survey. Some people did a home survey, the school did its own survey and the census comes here. But no-one has been interested in our animals before.”

**9**

**Village**: **Date**: 18 /3/ 17 **Time**: 2pm

Interviewer: Andy Translator: Jadu

**Location/ who else is present / what else is happening?**

3 men, 2 ladies, 1 girl. Outside house, people working paddy in front. Man of this house too busy in paddy to interview, neighbour turned up so we are interviewing him first instead.

**Name**: **Male/ ~~female:~~** **Age:** 43

**Your family and household**

**Who lives in your house?**

(Can we meet them please? What do they do? What do you want to do when grown up?)

Wife

Son: 23 company job (MRL) temporary work in a refinery

Daughter: 19 study HS final

**How is your family/ household supported?** (food, money, rent, etc).

Farm; paddy, mustard, veg for home

**What animals does your family/ household have? Why are your animals important?** (Can we see your animals please? Why do you keep them? How do animals help to support your household? )

Cow 2 Sell milk, calves also

Calf 3 Sell sometimes at 4 – 5 yrs, males only, keep females for more cows

Goat 5 Selling, never eating

Duck

Chicken 4 Sell chickens, eat eggs

Ox

Rent tractor when required

**What is a ‘good’ animal?** How do you know?

(Another man arrives and joins in )

Height, face- especially teeth- age, legs, good hooves, muscle- particularly legs, strong backside.

When buying a cow I take an experienced guy with me to help

**How do you know if an animal is healthy?** How do you know if an animal is unhealthy?

Thin (bad sign), a good cow should have good muscle.

A cow should be in good mood and eat well. A sick cow stops eating or does less eating.

Healthy cow is active all day, a sick cow is less active and lays down.

**What diseases/ problems are important for your animals?**

1. “Dingi- Phulla” or “Gol-Phulla”: (neck swelling) 1 or 2 animals, occurs after flood, die every time after 3-4 days, swelling under chin also dysentery- very watery
2. “Kura- patta” (broken hoof): Hoof falls off, new hoof grows back but flies lay eggs inside hoof, the back of the hoof separates and a gap appears. The skin between the toes can be affected too. Sometimes there are maggots. The animal eats less because it cannot walk.
3. “Pet- phulla” (fat belly) aka “Dhoka- dingra”: all die in 12 hours, goats and cattle, young and adult, one at a time, occurs after flood at grazing or in shed.
4. “Dysentry” (dysentery): Loose motion of goat or calf, mostly young animals, usually occurs after flood. Bloody dysentery- will die, otherwise survive but sometimes die.

**Can you teach me how you recognise the disease? Treat?** Prevent?

1. Pray. If worse can send for Dr, still may die.
2. Tie mouth so can’t lick foot. Phenyl on foot if flies lay eggs, never if no maggots. Wash wounds with KMnSO4.
3. No time for treatment.
4. Dr medicine , may survive, may die.

**How often do these problems occur?**

1. Long time ago
2. After flood 2016
3. After flood 2016
4. After flood 2016

**What things limit how much your animals give?** ( provide for your household?)

Not enough green grass available

**Young Animals**

Where do you keep your calves? Can we see please?

**How many of your cows/ buffs/ goats had a baby in the last year?**

2 cows, 3 calves- 1 each cow this year, 1 older calf (only one calf born last year)

**Where are those baby animals now?**

Here

**When a calf is born, can you teach me about the first day of its life?**

(When should it first drink milk (colostrum)? How much? What can you do to help it to be healthy? Who helps it?)

Take Pha-Hoo from cow, we take approximately half, about 1 litre, by milking all four teats half empty. The calf then drinks the other half of the Pha- Hoo. The calf drinks when it is about 30 minutes old.

**How much milk does an older calf drink and how often does it get it?** (Can you teach me how you do this? Who is responsible for this?)

Next milking at 10 – 12 days. Cow and calf together at all times until then.

After this cow and calf separated overnight. Release calf to go to cow, then catch calf again after a few minutes. Milk cow, then calf goes back to cow for the day.

Cow is milked once daily in morning.

My wife does this.

**Do your calves have any food apart from milk? Water?**( Where? How often? Who’s job?)

Start to eat about 1 month, start grazing. Calf should do this on it’s own. Cow goes grazing and the calf goes with her.

Taken water 3 times daily.

**How much milk does your family get each day from your animals?** (What do you do with it? When do you first milk a cow/ buff /goat for drinking?)

1.5 L per day (per cow?). Sell most, some for house.

**Adult Animals**

**Can you show me where your animals rest in the day? At night?**

Paddy day, shed night

**How do your animals get food? What**? (Who (family member) is responsible for this? Can we meet them please? How much land do you have? Can we see it?) **Where do your animals drink? How often?**

Evening rice straw and rice husk.

Water 3 times daily

My wife does this work.

**Did you vaccinate your animals last year? Why / why not?**

This year, yes. Usually we don’t use vaccine, I don’t really know why, we just don’t.

We only vaccinate when the government or some organisation (NGO) come to the village and do it.

**Do you de-worm animals? How? Why/ why not?**

Dr does it, only the calves with an oral medicine. Sometimes we see worms in the dung, we don’t do anything. We see them soon after treatment. Long flat worms- “Fita- pellu”

**Do you do anything else to help your animals?**

Clean shed

Wash cows every 3 – 6 months

**You live close to the forest reserve. Does this affect you?**

Crop raiding- elephants, boar, wild buffalo, (only occasionally rhino), parakeets.

**Animal health education**

**Is there anyone who can treat your animals?** (What would you like the vet doctor to know more about?)

Compounder in Sabzuri. Comes when needed.

**Would you like to learn more about animal health? Why?**

I would like tyo learn from a Dr or compounder how to treat my own animals.

**What problems are there for people trying to learn about animal health in your village?** ( Why? Which ways of learning about animal health would be good for you (village meetings, classes, leaflets, radio broadcasts, internet information, etc)?)

“Practical teaching. Meetings would be very useful. I could learn something from leaflets also.”

Insufficient Dr’s and resources. No one ever teaches us.

“Because it is for me I will have to manage time, and I will manage.”

**What changes could make your farm to more productive/ better?**

No changes.

“To produce more milk I would like to cross breed some of my animals.”

**What do you think is the future of this farm? (**Who will you pass your knowledge of farming on to?)

I don’t want my children to carry on the farm.

“In my younger age I did not receive things (education). For this reason I have provided for them education and other things. That is why I will be very happy if they get some jobs.”

**Anything else you would like to tell us?**

“If you provide us some training we can be very grateful.”

**10**

**Village**: **Date**: **Time**:

Interviewer: Andy Translator: Jadu

**Location/ who else is present / what else is happening?**

Home. Son, female friend and her daughter (approx. 9 yrs)

**Name**: **~~Male~~/ female:** **Age:** 33 years (initially forgot)

**Your family and household**

**Who lives in your house?**

(Can we meet them please? What do they do? What do you want to do when grown up?)

Self

Husband

Daughter 15 8 standard

Son 18 farming

**How is your family/ household supported?** (food, money, rent, etc).

Farming: paddy, mustard, Daal, veg- sell what family doesn’t eat

Daily labour

Weaving

**What animals does your family/ household have? Why are your animals important?** (Can we see your animals please? Why do you keep them? How do animals help to support your household? )

Cow 3 milk- sell and drink, calves

Calf 5 keep strong, sell weak

Goat 0

Duck 5 + 18 ducklings eat and sell ducks and eggs and make more ducks

Chicken 8-10 as above

Ox 2

**What is a ‘good’ animal?** How do you know?

Cow: height, strong body, any colour, udder size (big = more milk), eating well

Ox: Leg muscles, walking style, hump (big = good), big height, long tail (keep flies away)

Husband would buy new animal. He goes alone- very knowledgable

**How do you know if an animal is healthy?** How do you know if an animal is unhealthy?

Stop eating, mood change: bad mood, lethargic, lays down more

Touch animal- feel fever, hair stands up

**What diseases/ problems are important for your animals?**

1. “Dysentery”: goat and cow, any age- after flood- 1 or 2 animals- become well in 10 – 12 days if treated, die if not treated.

“One cow milking died the same day (in 2016) had been ill for 7 days, had received Dr treatment.”

1. “Kurra- patta”: after flood, spreads, cow and goat. Opening occurs at top of hoof (coronary band)- panful, stumble walk, sometimes hoof deformed or lost. Flies and maggots between claws. No other signs occur, keep eating normally.
2. “Pet-phulla”: whole belly distended- stop E/D/U/F, lasts 2d, some die, 1 or 2 animals affected after flood.
3. “Papora- Dhora” (goat rash): Hair falls, rough skin white colour, spreads to cover whole body, very itchy. Spreads from one goat to another. May die if untreated.

**Can you teach me how you recognise the disease? Treat?** Prevent?

1. Dr or “Mosundri” (leaf)- chop and give juice in a betel leaf, sometimes works.
2. Tie in mud. If maggots camphor (sprinkle powder) or tobacco (mix mix CaCO3 and pack wound)- effective.
3. Pray
4. Dr injection. Hadli juice effective against itch only.

**How often do these problems occur?**

1. 2016
2. After flood 2016
3. After flood 2016
4. 2013- 2014

**What things limit how much your animals give?** ( provide for your household?)

Tiger. Never killed one of my cattle. Killed the neighbours cattle.

Small jungle cats- eat ducks and chickens.

**Young Animals**

Where do you keep your calves? Can we see please?

**How many of your cows/ buffs/ goats had a baby in the last year?**

3 cows, 5 calves. All calved this year, 2 older calves here + 1 sold.

Cow died 2016 dysentery, calf died of no milk afterwards (was 7 -8 wks old)

**Where are those baby animals now?**

**When a calf is born, can you teach me about the first day of its life?**

(When should it first drink milk (colostrum)? How much? What can you do to help it to be healthy? Who helps it?)

Clean calf, give calf “Phao- Hoo” (15 – 20 mins) then milk phao hoo for house- 0.5L (udder not empty)

Help calf to stand and drink. Open mouth and put on teat. “It can be very difficult to feed new born calves.”

“If it drinks the first milk it will become strong and healthy. If it does not drink it will become thin and weak. It may die if it does not get the yellow milk.”

**How much milk does an older calf drink and how often does it get it?** (Can you teach me how you do this? Who is responsible for this?)

Don’t milk cow for 30 days, cow and calf together all the time for 30 days.

After 30 days- separate at night, after midnight. Milk cow at 7am, don’t milk empty- get 1.5 – 2 L

**Do your calves have any food apart from milk? Water?**( Where? How often? Who’s job?)

Grazing only, start to eat a little at 3 months.

Water 3 times daily

Wife responsibility.

**How much milk does your family get each day from your animals?** (What do you do with it? When do you first milk a cow/ buff /goat for drinking?)

1.5 – 2 L cow/ day. House and sale.

**Adult Animals**

**Can you show me where your animals rest in the day? At night?**

Paddy, shed

**How do your animals get food? What**? (Who (family member) is responsible for this? Can we meet them please? How much land do you have? Can we see it?) **Where do your animals drink? How often?**

Grazing

Rice straw- all year

Rice polis (uncooked) in water

Always salt in water

Water 3 x daily

**Did you vaccinate your animals last year? Why / why not?**

Yes. Many years, only do if Forest Dept or some organisation (NGO) do.

Prevents dz: Kurra patta, pet phulla, dysentery.

**Do you de-worm animals? How? Why/ why not?**

Tablets to calf at 6 m/o and 12 m/o, from Dr or pharmacy.

Yes we see worms (“Pelu”), small and flat

**Do you do anything else to help your animals?**

Clean shed daily

Smoke shed to prevent flies and mosquitos in summer.

**You live close to the forest reserve. Does this affect you?**

Crop raiding: elephant, boar. Occasionally elephants damage houses and raid grain stores.

**Animal health education**

**Is there anyone who can treat your animals?** (What would you like the vet doctor to know more about?)

Dr knows a lot.

**Would you like to learn more about animal health? Why?**

Yes. I can look after my own animals and help my neighbours.

**What problems are there for people trying to learn about animal health in your village?** ( Why? Which ways of learning about animal health would be good for you (village meetings, classes, leaflets, radio broadcasts, internet information, etc)?)

“If you teach us we can learn. Practical training by some experts would be very helpful to us. But I can learn from posters leaflets and flags too.”

“We don’t have the environment for learning here. At my younger age I didn’t want to learn about these things. Now I want to learn because if I learn I can keep my own animals healthy and more productive,”

Only 10% can read here (guess)

“Many people in this village are farmers and raise cattle and don’t go for study and those kind of things. Only younger generation is interested in study and college.”

**What changes could make your farm to more productive/ better?**

**What do you think is the future of this farm? (**Who will you pass your knowledge of farming on to?)

Children will carry on working farm

**Anything else you would like to tell us?**

**11**

**Village**: **Date**: 19/03/17 **Time**: 10 am

Interviewer: Andy Translator: Jadu

**Location/ who else is present / what else is happening?**

Outside house, wife, baby (belogs to another lady), son comes later

**Name**: **Male/ ~~female:~~** **Age:** 58

**Your family and household**

**Who lives in your house?**

(Can we meet them please? What do they do? What do you want to do when grown up?)

Self

Wife

4 sons 30, 27, 25 carpenters and farming, 22 farming

1 daughter in law 25

1 grand daughter 4

**How is your family/ household supported?** (food, money, rent, etc).

Farming: Paddy, mustard, black daal, sell vegetables: cabbage, brinjal, bottle gourd

“Mahori” village representative on Forest Dept labour committee- oversees work- receives same money as labourers.

**What animals does your family/ household have? Why are your animals important?** (Can we see your animals please? Why do you keep them? How do animals help to support your household? )

Cow 1 milk and dung 1L sale, feed calf and sale

Calf 2 sale

Goat 4 sale

Duck

Chicken

Ox

Cultivate: rent tractor

Ox killed by tiger 10 yrs ago, at that time tiger killed 13 cattle in 2 mths.

Want to get more ox, ox makes dung for compost, don’t have to pay tractor. Can’t afford outlay cost for ox (“money problem”).

**What is a ‘good’ animal?** How do you know?

Legs, walking style, health, size

“I don’t have that knowledge”

“I only look at its legs and its walking style and I guess if it is a good animal.”

“I take an experienced man from the village when I go to buy cattle and I listen to what he says.”

**How do you know if an animal is healthy?** How do you know if an animal is unhealthy?

Bad mood, dripping saliva, ears down, difficulty breathing

**What diseases/ problems are important for your animals?**

1. “Saboka”- after flood “Kura- Patta” – same problem. This is our main problem. Wound comes in feet- blood comes out – maggots come. Stops eating, tongue infection can occur. Fever also. Spreads from one to many. Not always every year in every animal. If animal not noticed and not treated death is possible (death is due to lack of vitamins in the body). With good treatment full recovery in 3- 4 months. Abortion can occur (asked about this) but is not common; cow and calf can both die.
2. “Gol- Phulla”- swelling of neck- noise “Grrrr”- chin and neck to brisket swelling (oedema?). Goat and cow, young and old, some animals (sporadic?) after flood. Recover if Tx, otherwise die.
3. “Pet – phulla”- whole belly big no D/E/U/F, difficulty breathing. Usually 1 or 2 animals but in occasional homes all animals. Starts after flood and goes on for 3 – 4 months. Recover if treated, otherwise die, small chance of survival. Goats and cows.
4. “Go – Basanta”- Pox in cows – infection in tongue, saliva drips. Iinfection in small patches whole body and hair falls out of patches. Red wounds below/form, flat, skin peels. Itchy and animal rubs body. Doesn’t affect feet. Spreads from 1 to 3 animals, not whole village. Takes 5 – 6 months to get well. Occurs after flood. If whole body affected- stops E/D and may die of weakness.

**Can you teach me how you recognise the disease? Treat?** Prevent?

1. Permanganate solution and use to wash wound, apply Himax. Dr gives treatment but no injections.
2. No treatment. Vaccinate.
3. Crush ginger , mix with black salt, wrap in a banana leaf and give by mouth (as a bolus) – sometime works. If not getting better see Dr. Dr may anus and give IV drip. My cow still died but my neighbour’s cow survived.
4. No local treatment. Dr put ointment on the wound and gives injection.

**How often do these problems occur?**

1. 2009
2. Never happened to my cow
3. Cow died 2012
4. Never at this house.

**What things limit how much your animals give?** ( provide for your household?)

Less availability of grass.

Unable to buy good quality foods for animals. We don’t have that kind of money so we are unable to provide good nutrition. Sometimes I buy food, but not enough for all animals and this reduces productivity. Husk and grain that I mix in cooked food.

Animal nutrition is biggest problem from March to July.

“March to April (before flood) is the best time to vaccinate animals. Private vaccine is better than Govt. If vaccination is done in proper time saboka never occurs. Vaccine also for Gol phulla, Pet phulla and Go Basanta. Used to consult Dr, but will always vaccinate if vaccine available, but because of poor availability of vaccine Forest Dept don’t always come.

**Young Animals**

Where do you keep your calves? Can we see please?

**How many of your cows/ buffs/ goats had a baby in the last year?**

1 cow, 1 calf (2016), 1 older calf (2015)

**Where are those baby animals now?**

Here

**When a calf is born, can you teach me about the first day of its life?**

(When should it first drink milk (colostrum)? How much? What can you do to help it to be healthy? Who helps it?)

If dystocia- farmer correct- most people know this “All people in this village know about those things because the veterinary doctor is not always available.

Last delivered a calf here in 2007/8. “My wife did it, she knows a lot more about these things than me.”

Clean calf (womans job), clean face and nose, sometimes blow air in nostrils. Take calf out of shed to a sunny place if born in the day. Stands after 1.5 – 2 hours, take to cow and help to feed “Open mouth and put to teat, gently rub it’s head and then it starts to suck on its own. Sometimes you put your own finger in it’s mouth to encourage it to suck.

Calf drinks first, then we take some Phae Ho. Calf drinks for 2- 3 minutes. Take about 1 L depending on cow size, big cow, take more. After another 1 – 1.5 hour allow the calf to drink again. “Someone looks after the calf for the first 12 hours to check that it is drinking” (wife says). Calf is full in 2 minutes.

Next milking 10 days. Calf and calf are together all that time. Once milking, separate the calf from the mother at 1 or 2 o’clock – someone has to wake up. At 8 am milk cow- let calf out to drink first – 10 minutes – then milk cow- 1 -1.5L. The cow that died used to give 2.5L

**How much milk does an older calf drink and how often does it get it?** (Can you teach me how you do this? Who is responsible for this?)

Wife is in charge of calf and milking things.

**Do your calves have any food apart from milk? Water?**( Where? How often? Who’s job?)

Give nothing while still drinking milk. Starts to graze 5 – 6 months.

Water 1x or 2x daily, noon and evening, wife does. “Differs from house to house but 80 – 90% of women do this work. Tie cows in the paddy or shed is usually a man’s job. All other things are done by women. Man usually is mostly engaged with cultivation.”

“Man work is mostly hard work like cultivation, fetching heavy things or if the house is broken. Things women can’t do.” (Hard work or heavy work?) “Hard work.”

**How much milk does your family get each day from your animals?** (What do you do with it? When do you first milk a cow/ buff /goat for drinking?)

Sell 1 L daily.

0.5L for children and tea, this is sufficient. We have stopped drinking milk tea because it (milk?) gives older people gas problem.

**Adult Animals**

**Can you show me where your animals rest in the day? At night?**

Paddy day, shed night

**How do your animals get food? What**? (Who (family member) is responsible for this? Can we meet them please? How much land do you have? Can we see it?) **Where do your animals drink? How often?**

Rice straw (whole year) and cooked food- rice husk, vegetables and vegetable leaves.

During flood time cooked food includes chopped banana tree.

Water 2x daily, noon and evening.

Man and woman’s job, whoever is available.

**Did you vaccinate your animals last year? Why / why not?**

Yes. Since 2001. Prevents Dz.

Pet-phulla, Gol-phulla, Saboka, Bo-santa: vaccine different for different diseases.

“I always keep in touch with Forest Dept and use vaccine if available. If vaccine is not available sometimes we go to the pharmacy and buy it privately and do it ourselves.”

**Do you de-worm animals? How? Why/ why not?**

Yes, tablets- not frequently- at 1 year and after 2 or 3 years. Goats and calves. “Usually goats aren’t affected, it is mostly the calves. (Do you see worms in dung?) Son says: yes I have seen in the village (please describe) fat white worms in calf dung (show photo of T vit in dung) yes, like that.

**Do you do anything else to help your animals?**

**You live close to the forest reserve. Does this affect you?**

Tiger killing cows

Crop raiding and destruction of houses by elephant. Rhino, deer and wild boar also raid crops.

**Animal health education**

**Is there anyone who can treat your animals?** (What would you like the vet doctor to know more about?)

“He comes when we call. I can’t say he is a good doctor. He is good for small problems but if the case is major sometimes the animals die.”

“It would be good for us if he knew a lot.”

**Would you like to learn more about animal health? Why?**

“Yes. Doctor takes some fees. If I know these kind of things I would not have to give money to anyone. I could buy tablets and treat myself.”

**What problems are there for people trying to learn about animal health in your village?** ( Why? Which ways of learning about animal health would be good for you (village meetings, classes, leaflets, radio broadcasts, internet information, etc)?)

“Because I am growing old day by day, theory will not be enough for me. I need practical training. But for my sons, they can learn. They will benefit from theory and practical training.”

**What changes could make your farm to more productive/ better?**

Because of flood, this ground is not high enough. I want to buy improved quality cows but because of flood problem I am not buying. I want to make this ground higher, then perhaps I will buy.”

I want to make a good shed for the cows and then I can buy good quality cows and make more milk. I want to make a good shed with some metals and then I can protect them from the tiger.

**What do you think is the future of this farm? (**Who will you pass your knowledge of farming on to?)

“I am teaching my sons and they will carry on farming here. I am teaching all of my sons to be independent by farming, carpentering, and any kind of thing that they can do to make their lives better.”

“I am doing village politics but I don’t want my sons to become politicians.”

**Anything else you would like to tell us?**

“It is good that you are writing down everything that I am saying. You are actually listening to what I am saying.”

“I am very grateful that you have come here. We are hoping to get more from you than this survey. Not in a physical manner but teaching us so we can help ourselves. We are poor people raising cows and ox. Our biggest problem here is the tiger. Tiger causes a lot of damage. If it kills a cow or an ox worth Rs 30,000 how can we deal with that. We need help from Forest Dept or some NGOs to deal with this problem.”

“At first I went into politics unwillingly because a Party Member (Congress) came to this village and said that we need someone to represent your village so I went with it to represent my village. Now I release that it’s not worth it. I am not getting anything from this politics thing. I have to spend my own money every time I go somewhere, they give me nothing.”

**12**

**Village**: **Date**: 19/3/17 **Time**: 12 pm

Interviewer: Andy Translator: Jadu

**Location/ who else is present / what else is happening?**

Home. Father and a lad present

**Name**: **Male/ ~~female:~~** **Age:** 35

**Your family and household**

**Who lives in your house?**

(Can we meet them please? What do they do? What do you want to do when grown up?)

Self: carpenter

Mother

Father

Brother: 30, carpenter

Brother 26

Wife

Sister in law

Daughter: 6

Son: 1.5

Niece: 4

**How is your family/ household supported?** (food, money, rent, etc).

Farming: paddy, mustard, vegetables- home only

Carpenter

**What animals does your family/ household have? Why are your animals important?** (Can we see your animals please? Why do you keep them? How do animals help to support your household? )

Cow: 1. Milk and calves

Calf: 2 (1 older)- sell at 4 years.

Goat : 10. Sell, never eat, but we would if we wanted,

Duck : 10. Eat, never sell. Eggs- some for eating, some for more ducks.

Pigeon: 6. Eat never sell

Chicken

Ox: 2

**What is a ‘good’ animal?** How do you know?

Ox: Legs- muscle, tells you their strength. Long legs will grow more big size.

Tail- long keep away flies and mosquitos

Cow: Fat (healthy) belly, height- but body size more important. Big udder.

**How do you know if an animal is healthy?** How do you know if an animal is unhealthy?

Mood- low mood if sick, ears drooped. Belly pain- looks round.

**What diseases/ problems are important for your animals?**

1. No name new Dz: Occurred for the first time in January. Any age, leg swollen, sound – gas – when touched. Stops eating, lays down, dies in 24 hr. Leg feels wet. Can be all legs or whole body. One animal at a time.
2. “Kura- Phatta”: stumble, hoof falls. 1 month recovery if Tx. Maggots may come. If no Tx- hoof may fail. All animals, every year, cow’s don’t abort.
3. “Gol- Phulla”: swelling under chin- cow and goat- remain healthy- occurs at any time of year.
4. “Bohonta” or “Basanta”: cow, patches all over body, hair falls, patches all over, then skin comes off, patches join, eat less. Not a/w animal colour. Itchy, cow always licking. Death is rare, recover in 2 – 3 months.
5. No name disease; ducks- saliva comes from mouth sometimes all flock dies- spreads from one to another. Stop walking, sleep a lot, die. Never survive, die in less than 24 hr.

**Can you teach me how you recognise the disease? Treat?** Prevent?

1. No Tx
2. Tie in mud. Maggots- local medicine- herbal, don’t know what – once daily for 5 days, cow eats, very effective. Himax to prevent.
3. Tie “leteku” (daal straw) around neck. Prayer. “Jorphuka” – special man, comes to home – salt in hand, says prayer and spreads salt on cow. Give man something, not always money, may give betel leaf and nut. Effective.
4. Prayer. Light incense sticks, call village folk (usually a few people from nearby houses come), give people “prasad” (temple sweets) and all people pray.
5. No Tx.

**How often do these problems occur?**

1. Jan 2017
2. After flood 2016 in this house
3. Never happen in this home.
4. Rainy season 2016
5. After flood 2016

**What things limit how much your animals give?** ( provide for your household?)

Availability of grass

After flood- no grass in this area.

**Young Animals**

Where do you keep your calves? Can we see please?

**How many of your cows/ buffs/ goats had a baby in the last year?**

1 cow, 1 small calf, 1 large calf

**Where are those baby animals now?**

Here

**When a calf is born, can you teach me about the first day of its life?**

(When should it first drink milk (colostrum)? How much? What can you do to help it to be healthy? Who helps it?)

Call Dr if dystocia

Look after calf, if he can’t stand or suck, help him. If unable to drink- feed with bottle (100 – 200ml) if not fed in 2 hours.

Milk out 1^st^ Phae- Ho as soon as cow stands up. Don’t milk empty, if 2 L cow- take 1L Phae - Ho.

Give cow food including pepper- boiled veg to strengthen cow.

**How much milk does an older calf drink and how often does it get it?** (Can you teach me how you do this? Who is responsible for this?)

Next milking at 3 days – throw milk in river, and at 5 days throw in river. People avoid stepping on milk. My father does this, it is religion (seems unhappy about this). Repeat every 2 days until 11 days. Milk once daily until calf is grazing, then milk twice daily.

Increase separation time gradually, separate at 3 am, 2 am, 1 am.

At 8 am- calf drinks for 2- 3 mins- then milk 1.5L- rest for calf.

**Do your calves have any food apart from milk? Water?**( Where? How often? Who’s job?)

Starts to graze at 3 m/o

If belly goes fat (between 4 – 12 mths old) – know worms present. Give tablets.

Seen fat worms.

**How much milk does your family get each day from your animals?** (What do you do with it? When do you first milk a cow/ buff /goat for drinking?)

Once daily: 1 – 1.5L daily – all for house, sell none.

Twice daily: 2.5L daily

Father does.

**Adult Animals**

**Can you show me where your animals rest in the day? At night?**

Graze paddy- tied, shed at night.

**How do your animals get food? What**? (Who (family member) is responsible for this? Can we meet them please? How much land do you have? Can we see it?) **Where do your animals drink? How often?**

Cook food- vegetables, wheat grains, rice polis

Jaggery after giving birth. Father does.

Water: noon and evening- father does.

**Did you vaccinate your animals last year? Why / why not?**

Yes. 10 years. Prevents Dz spreading in national park. Stop animals going sick- Kura- phatta, Pet- phulla, Leg Dz (not sure what Dz’s). Forest Dept did last year.

**Do you de-worm animals? How? Why/ why not?**

Calves 4 – 12 mths if belly big.

**Do you do anything else to help your animals?**

Give food at proper time.

Change grazing position.

**You live close to the forest reserve. Does this affect you?**

Crop raiding and damage done by wild animals: elephant, rhino wild boar, deer.

There are no positive things about having the park here.

**Animal health education**

**Is there anyone who can treat your animals?** (What would you like the vet doctor to know more about?)

Very good Dr, but sometimes cannot save 1 or 2 animals. 3km away. Have to pay according to medicines provided- affordable.

**Would you like to learn more about animal health? Why?**

“Yes, so I can treat my own animals and teach other people in the village also.”

**What problems are there for people trying to learn about animal health in your village?** ( Why? Which ways of learning about animal health would be good for you (village meetings, classes, leaflets, radio broadcasts, internet information, etc)?)

I can only learn by practical teaching. I can read, but I can’t learn by reading a book.

Posters and visual learning (asked about this), some difficulties may arise.

Yes, I can manage time to learn.

**What changes could make your farm to more productive/ better?**

Make proper, clean shed for animals.

Make it clean to prevent sickness.

**What do you think is the future of this farm? (**Who will you pass your knowledge of farming on to?)

I want to make this farm bigger, but I cannoy because of my economic condition.

“I want my children to have good jobs, not farming.”

**Anything else you would like to tell us?**

“No. I have nothing else to say.”

**13**

**Village**: **Date**: 19/3/17 **Time**: 1.30pm

Interviewer: Andy Translator: Jadu

**Location/ who else is present / what else is happening?**

Inside house, old man and small son also present, then brother arrives and joins interview. Vegetarian family.

**Name**: Two brothers **Male~~/ female:~~** **Age:** 52

**Your family and household**

**Who lives in your house?**

(Can we meet them please? What do they do? What do you want to do when grown up?)

“I learned about raising cows from my father. Most people in this village do not know about raising cows.”

Self

Wife

Son 10

Grandson 6

(Has 4 daughters, married, living other places)

I wants my son to be well educated and have a job. I am also teaching him morals and manners. He is getting good marks in school and his teachers are looking after him.

**How is your family/ household supported?** (food, money, rent, etc).

Farming: paddy, dhania, daal, (previously mustard, went on daal cultivation training course)

Carpenter (for 30 years)

**What animals does your family/ household have? Why are your animals important?** (Can we see your animals please? Why do you keep them? How do animals help to support your household? )

Cow: 1 gives milk and calves

Calf : 3 keep best ones and sell others. Haven’t sold one for 15 years. (how does this add up?)

Goat : 2 Gave to other people to raise (“Adhi”) 50/50 split profit.

Duck

Chicken

Ox: 0 Prevoiusly had 2 but sold as insufficient grass

Now I use rent tractor. I will but one ox when my older calf is big enough to use in the field. I prefer to work with ox than tractor. (Why?) The production is better when you use ox compared with tractor. ((Why do you think that is?) I don’t know why.

I sell my ox when they are old, or if they have some problem, like fever. I sold a cow that was not getting pregnant.

**What is a ‘good’ animal?** How do you know?

Cow: short height, fat round belly, soft hair, no horns.

Ox: Big face, medium size horn, strong front side, long tail, round front (when viewed from front)

Prefer red or white animals, but this is secondary consideration.

**How do you know if an animal is healthy?** How do you know if an animal is unhealthy?

Mood. Sick cow is sad, healthy cow is energetic and in good mood. Sick cow is laying down, laying down is not a healthy sign, it has less activity and does less eating and drinking.

**What diseases/ problems are important for your animals?**

1. “Sikora” – ticks- weakens animal. If very many ticks animal can die. Occurs in rain and hot season. Animal also has dandruff.
2. “Basanta” or “Bohonta”: summer problem. Small patches on skin, hair comes out, wounds begin shallow and get bigger.
3. “Saboka”: infection in tongue and foot, affects all animals including goats. Starts in skin between toes, hoof affected, can separate, maggots may come in hoof.
4. “Dysentery”: Cow and goat, affects many animals, occurs after flood. Eye Dz may come later (linked?). Die without treatment.
5. “Pet- Phulla”: whole belly swells, occurs after flood. Animal dies unless it passes dung.
6. New type “Pet –Phulla” body shakes, neck swells, small boils inside neck (can feel them), head may be crooked. Tongue extended, takes a long time to breathe (increased respiratory effort?) Can’t stand. Dies in 7 or 8 hours. Occurs after flood.
7. “Dohoka- Dhingira”: Outside of neck swells, stops D/U, Rapid death. Occurs during winter season, usually between midnight and dawn. Only happens to strong cow/ox, it is related to blood poison.
8. “Forget name”: swelling under chin, goats mostly, cows also, after flood, one at a time, die because they stop eating- takes about a month.
9. “Papora- Dhoka”: goat skin Dz- hair falls out, skin is rough and white. Itchy. Spreads- can cover whole body. Spreads from one animal to another. Don’t die.
10. “Letekula”: eye disease, comes after dysentery. Eye turns white, water (fluid) comes from eye, twitchy eyelid ( blephrospasm), if not treated can go blind
11. “Okoni” – fleas. (Didn’t elaborate).

**Can you teach me how you recognise the disease? Treat?** Prevent?

“All local medicines are temporary. You must do to the doctor for treatment.”

1. Remove tick
2. Pray. Gets well in 3 – 4 days. Prevent by avoiding mud.
3. Maggots- use crop pesticide, dissolve in water and spray in wound.
4. Preserved lemon and call Dr.
5. “Cow has to pass dung or it will die. If you scare cow, cow will pass dung and become well. I scare the cow like this (man demonstrates how he scares cow with jumping motion and scary face).”
6. No treatment dies rapidly.
7. No treatment possible, dies to fast.
8. Dr Treatment
9. Dr injects
10. Haldi in eye. If not better- salt in person’s mouth and spit into eye. Hinges plant (ink plant)- crush plant for juice and apply juice to eye- repeat 3 times. If still not well tie letekula stem around neck.

**How often do these problems occur?**

1. Now ( March 2017)
2. Hot season 2015
3. After flood 2013
4. After flood 2016
5. Long time ago
6. Neighbour 3 years ago
7. Neighbour 3 years ago
8. After flood 2014
9. 2016
10. Never to my cattle.

**What things limit how much your animals give?** ( provide for your household?)

**Young Animals**

Where do you keep your calves? Can we see please?

**How many of your cows/ buffs/ goats had a baby in the last year?**

1 cow, 1 calf

**Where are those baby animals now?**

Alive, here.

**When a calf is born, can you teach me about the first day of its life?**

(When should it first drink milk (colostrum)? How much? What can you do to help it to be healthy? Who helps it?)

If dystocia occurs- fetch …..’s wife. Usually it is women who help animals to be born, they understand this work.

Pull legs and tail (makes legs strong), Blow air in ear, eye, nose and mouth (clears blockages), male calf- press hump (makes bigger).

Protect calf from other animals.

If winter make fire to keep warm

Clean calf with warm water; give cow hot water to drink.

Give cow bamboo leaf and sugar cane leaf to eat- placenta falls quickly.

Tie rice straw through calf’s mouth after pulling legs, leave for 10 minutes.

When calf tries to stand- help it to feed from mother.

Phay Hoo- calf drinks 15 -20 minutes then milk cow for Phay Hoo for house- 0.5L, sometimes less. Estimate amount of milk from the size of the cow- half for the calf.

Don’t milk cow for 7 days, cow and calf together the whole time.

**How much milk does an older calf drink and how often does it get it?** (Can you teach me how you do this? Who is responsible for this?)

After 7 days, separate cow and calf at midnight and increase this time over some days. In morning take cow out and tie in a clean place, untie calf- let down occurs- allow calf to suck for 2 minutes then remove calf- milk cow- take half milk, leave the rest for the calf.

Calf must drink as much as possible, otherwise becomes weak.

**Do your calves have any food apart from milk? Water?**( Where? How often? Who’s job?)

After 6-7 months: rice husk and rice polis in water with salt

Water 3 times daily.

**How much milk does your family get each day from your animals?** (What do you do with it? When do you first milk a cow/ buff /goat for drinking?)

1.5L / cow / day. Sell 1L.

Wife does milk and calf jobs.

**Adult Animals**

**Can you show me where your animals rest in the day? At night?**

Day: tie in paddy, Night: shed

**How do your animals get food? What**? (Who (family member) is responsible for this? Can we meet them please? How much land do you have? Can we see it?) **Where do your animals drink? How often?**

Left over veg and veg leaves, rice husk, rice polis, grain polis with water and salt

Water 3 times daily

Husband and wife both do

Flood time: chopped banana tree with rice husk and rice polis and rice straw.

**Did you vaccinate your animals last year? Why / why not?**

Yes, last 10 years.

Forest Dept tell us vaccination can get rid of Saboka and prevent spread to wild animals.

Also Basanta and Bohonta.

**Do you de-worm animals? How? Why/ why not?**

Yes, tablets from Dr, cow and calf and goats treated.

**Do you do anything else to help your animals?**

Vitamin tablets sometimes.

**You live close to the forest reserve. Does this affect you?**

I am very positive about the forest. There are many varieties of wild animals and many people come to see them. Many people are engaged in this work. We are very lucky because the rhino is the pride of Assam.

“I have no problems with the wildlife. God made all animals equal and all have a right to live. If sometimes animals venture out of the park and damage crops we receive compensation. It is not a big deal for me.”

“People have become very materialistic in this time. They are not loving the wild animals like they did in ancient times. If we can teach people to love all animals it make a really big difference, and to me also, it will make me very happy.”

**Animal health education**

**Is there anyone who can treat your animals?** (What would you like the vet doctor to know more about?)

Dr in Sabzuri 4km away and in Kanduli Muri 1.5 Km away. Good Drs and they come when they are called.

**Would you like to learn more about animal health? Why?**

I want to learn to treat animals and to raise in a better manner.

**What problems are there for people trying to learn about animal health in your village?** ( Why? Which ways of learning about animal health would be good for you (village meetings, classes, leaflets, radio broadcasts, internet information, etc)?)

Can read books but some things can be learned only from practical training. Posters and leaflets are also helpful

Lack of resources, the flood. I have to work hard to send my boys to school so time is limited.

**What changes could make your farm to more productive/ better?**

**What do you think is the future of this farm? (**Who will you pass your knowledge of farming on to?)

I want to make farm bigger, buy jerseys with more milk production and get high quality goats.

Food will be available for this but we will have to prepare well for the flood.

“I think my son will carry on farming. He likes to copy me and work the farm and he asks many questions and in anxious to learn.”

**Anything else you would like to tell us?**

**14**

**Village**: **Date**: 20/ 3/17 **Time**: 10am

Interviewer: Andy Translator: Jadu

**Location/ who else is present / what else is happening?**

Outside home, forest staff friend (30), niece, nephew

**Name**: **~~Male~~/ female:** **Age:** 34

**Your family and household**

**Who lives in your house?**

(Can we meet them please? What do they do? What do you want to do when grown up?)

Self

Mother

Brother 40

Sister in law 35

Nephew 20 (HS 1^st^ year)

Niece 18 (HS final year)

**How is your family/ household supported?** (food, money, rent, etc).

Farming: paddy, mustard, veg (sell and house)

Weaving

Occasional labour

**What animals does your family/ household have? Why are your animals important?** (Can we see your animals please? Why do you keep them? How do animals help to support your household? )

Cow: 2 calves and milk

Calf: 2 keep females to increase number, sell male at 3-4 years- sell for money problem or tiger problem.

Goat: 9 sell, occ eat (Bihu, Pooja)

Duck: 4 (Haa) selling

Chicken: 20 eating and selling and making more chickens (mostly eating)

Ox: 2 (Halua) Use from 3- 4 years, keep for (or until?) 7 -8 years then sell. If good healthy male calves then train themselves, otherwise buy. Training ox is hard, brother does with help from experienced men in village.

**What is a ‘good’ animal?** How do you know?

Healthy.

Cow: Length and height of cow, soft hair, round belly.

Ox: I don’t know much

**How do you know if an animal is healthy?** How do you know if an animal is unhealthy?

Stop grazing, laying down. By seeing I just know.

**What diseases/ problems are important for your animals?**

1. “Kura- Paata”: unable to walk, wound between toes, sometimes right around top of hoof. When starts, won’t walk, no blood, after 3- 4 days wound and blood, lasts more than a month. If they use Dr medicine (?). Might cause abortion (asked directly about this). All animals- cow, a few goats. June, July, August- after flood.
2. “Pet- Phulla”: Belly fat- sometimes one side or whole body. Cow stops eating. Cow, ox, goat, sometimes die if not treated. Mostly local treatment. One animal at a time, at any time of year, most happen in the paddy field- eating something wrong, not sure what- weeds?”
3. “Hagoni” (dysentery)- goats and cows, spreads from one to another, mostly happens after flood (cows?) in goats not related to flood. Old animals. Watery, sometimes pale, mostly die- cow and goat. “Farmers are scared when they see disease because they think that the animal will probably die.”
4. “Kandh Singa”: Hump wound (“Broken hump”) Mostly ox, rarely occurs in some cow. One or many, worse in cultivating season- June, July, December, January. Yoke (“Juloli”) causes- sharp/ rough edges injure skin from constant rubbing- yoke has to be very smooth. “Make best effort to make it free from sharp edges, this will reduce incidences.” “Sometimes maggots come.” Body shape also involved, so some animals are more susceptible.
5. “Papora Dhora”: rashes in goats. Winter season. Mostly goats, same rashes on cow occasionally. Hair falls, sometimes skin falls off too, skin is white, thick and rough in goat. Starts on belly if not noticed may die as spreads to whole body. Spreads between goats, cow one or two only. Itchy. People- itchy problem but different.
6. “Dhingi Fulla”: swelling under chin, spreads down a bit. Cow and goat. Hot season. One animal at a time. No other signs. Become well in 2 – 3 days, get thin, eat less. Not related to grazing animals
7. “Beculla”same as Dhingi Fulla.
8. “Basanta” or “Bohonta”: April, May, June. Cow and goat, itchy. Pox- small wounds anywhere, hair falls, skin falls, red colour.

**Can you teach me how you recognise the disease? Treat?** Prevent?

1. Wash with KMnO4 solution, apply Himax^TM^, apply tobacco if maggots.
2. Preserved lemon: 3-4 years old, give whole lemon wrapped in banana leaf and push down throat if animal doesn’t want to eat. Making preserved lemon- salt and put in jar dry. Also: Black salt with water- open mouth and pour in. Also: ganja leaf- chop and wrap in banana leaf.
3. Dr medicine, no local treatment.
4. Apply Himax^TM^, if maggots Dr medicine and/or tobacco. Clean wound then rub tobacco and CaCO3 to a fine powder and apply twice daily for 2 – 3 days- effective.
5. Neem in boiling water- wash animal 3 times daily for 7 – 10 days- if not well call Dr- injection- effective if given early, later on animal may die. Inject animal, wait 3 days- observe- if not itchy no further treatment.
6. No Dr Treatment- tie live house frog around neck- leave there until frog dies and animal becomes well- then cut string.
7. See above
8. Dr prescribes ointment- animal gets better.

**How often do these problems occur?**

1. After flood 2016
2. Before flood 2016
3. After flood 2016
4. One ox continually has a wound.
5. 2014
6. Long time ago
7. See above
8. 2016

**What things limit how much your animals give?** ( provide for your household?)

**Young Animals**

Where do you keep your calves? Can we see please?

**How many of your cows/ buffs/ goats had a baby in the last year?**

2 cows, 2 calves

**Where are those baby animals now?**

**When a calf is born, can you teach me about the first day of its life?**

(When should it first drink milk (colostrum)? How much? What can you do to help it to be healthy? Who helps it?)

If dystocia- call Dr., no experienced men in this area.

Clean cow, clean calf, clean navel with coconut oil, watch that no birds peck at navel cord.

Feed cow- cabbage or any available veg, rice husk.

Observe carefully and check that calf is drinking.

Phaa Hoo: before calf drinks milk out 1L from cow using all 4 teats. Make Phaa Hoo sweets, make also a lucky string of Phaa Hoo beads and put round cow and calf neck. Calf drinks half after cow is milked. Cow is milked 1 hour after calf is born, calf first drinks at 1.5 hours old.

Concerned about diarrhoea so prevent calf from drinking “Not drinking first yellow milk prevents calf from having diarrhoea.”

**How much milk does an older calf drink and how often does it get it?** (Can you teach me how you do this? Who is responsible for this?)

Next milking after 10 days, calf is fully drinking for 10 days, cow and calf together, cow is grazed but not tied when she has young calf.

Milked once daily for first month, then twice daily. Separate cow and calf at 2am, in morning let calf drink for 3 minutes then milk cow for 1 – 1.5L, some milk left in udder for calf.

**Do your calves have any food apart from milk? Water?**( Where? How often? Who’s job?)

Start grazing at 1.5 months old.

Give water at noon and evening.

Self or mother does.

**How much milk does your family get each day from your animals?** (What do you do with it? When do you first milk a cow/ buff /goat for drinking?)

1 - 1.5L cow/day, sell at little

If milking twice daily- nearly 2L

I milk the cow

**Adult Animals**

**Can you show me where your animals rest in the day? At night?**

Shed night, paddy day

**How do your animals get food? What**? (Who (family member) is responsible for this? Can we meet them please? How much land do you have? Can we see it?) **Where do your animals drink? How often?**

Cooked food- veg, rice water, rice husk, salt

During flood- banana tree chopped, elephant grass, rice straw, weeds from wetland.

Water 3- 4 times daily in flood, other times twice daily.

Self and mother do.

**Did you vaccinate your animals last year? Why / why not?**

Yes. 10 years. Prevent disease- Hagoni, Kura patta, Pet fulla.

**Do you de-worm animals? How? Why/ why not?**

No.

“Pel- lou”: worms

“Nee- mok”: salt

**Do you do anything else to help your animals?**

Concentrate mixture (powder)- give to lactating cows to produce more milk. Cooked like rice husk, smells slightly like yeast. 200- 300g mixed with veg or rice husk once daily.

**You live close to the forest reserve. Does this affect you?**

Crop raiding- wild animals, elephant, rhino

Tiger killing cattle. 4 animals- 3 cows and 1 calf (3 year old). Last time May 2014, occurred in shed- saw tiger and scared tiger away. WWF/ Forest Dept compensation Rs4000 (WWF Rs2500, FD Rs1500) per cow. Took 6 months to pay, Rs20,000 total. Bought new cow.

New cow Rs 15k, Ox Rs 30K – 40K/ pair or >15K single.

**Animal health education**

**Is there anyone who can treat your animals?** (What would you like the vet doctor to know more about?)

Dr 5km away, comes when we call if he is available. He is a good Dr.

**Would you like to learn more about animal health? Why?**

“Yes. If I could do primary care (first aid?) by myself and then I wouldn’t have to wait until the Dr comes.”

**What problems are there for people trying to learn about animal health in your village?** ( Why? Which ways of learning about animal health would be good for you (village meetings, classes, leaflets, radio broadcasts, internet information, etc)?)

“I can learn from books, but I also have to learn practically. Actually, in our place there is no government training and we don’t have any resources,”

“Yes, I can manage time to learn, but it will be difficult for me in flood season.”

**What changes could make your farm to more productive/ better?**

“If I got good blood (breeding) animals then my production will be more. I want to increase my number of cows and goats.”

**What do you think is the future of this farm? (**Who will you pass your knowledge of farming on to?)

“I will continue and then the next generation will take over. It continues from generation to generation. We have always lived here.”

**Anything else you would like to tell us?**

“If training can be provided it will make a difference to us.”

Nose roping: “ Tie the animals legs together and push it over, grab it’s head and piece nose. The spike pulls the rope through. We use a bamboo spike. This boy has done it. Pushing down a bull is hard, it takes three or four people to do it.”

Castration: “We get the Dr to attend the bullock. He uses a clamp (burdizo) and gives injection. We don’t use the village people for this.”

**14**

**Village**: DFP **Date**: 20/ 3/17 **Time**: 2pm

Interviewer: Andy Translator: Jadu

**Location/ who else is present / what else is happening?**

At home with husband

**Name**: **~~Male~~/ female:** **Age:** Thinks for a long time. “Maybe 45? I have forgotten.”

**Your family and household**

**Who lives in your house?**

(Can we meet them please? What do they do? What do you want to do when grown up?)

Self

Husband

Son 30 Carpenter

Son 27 Carpenter

Daughter in law 27 Shop

Daughter in law 26

Grandson 5 I want them to have good jobs like doctor. Boy says policeman

Grandson 4.5

**How is your family/ household supported?** (food, money, rent, etc).

Farming: paddy, mustard, veges- mostly for eating, occasionally sell

Shop

Carpentering

**What animals does your family/ household have? Why are your animals important?** (Can we see your animals please? Why do you keep them? How do animals help to support your household? )

Cow: 2 calves and milk- for house and sale

Calf: 2 keep females if we can, males always sold. We sell especially if we need money. It is like a cow bank

Goat: 0

Duck: 7 (Haa) eat and eggs for selling, eating and more ducks

Chicken: don’t keep now as we have ducks

Ox: 2 (Halua) Cultivation. Buy or use our own. My husband trains the ox.If he buys an ox he goes alone, he is very knowledgable. Buy the ox when it has two big (adult) teeth, start work (or sell on ox calf) when it has 6 adult teeth. Sell ox when it is about 10 years old. We guess the age by looking.

**What is a ‘good’ animal?** How do you know?

Cow: Legs should be long, belly round, long tail and big udder. Cow with big belly can eat more and will give birth to a strong calf.

Ox: Strong legs, long legs, good hooves- perfect shape- neat and tight. Horns should go up not out.

**How do you know if an animal is healthy?** How do you know if an animal is unhealthy?

Physique, dung- watery or very smelly, stops eating, change of behaviour, disease.

**What diseases/ problems are important for your animals?**

1. “Basanta” or “Bohonta : Changed behaviour- stop laying down, hot body; touch cow- if hair falls- assume bohonta
2. “Pet- Phulla”: Big belly and harsh breathing, fall down, lift tail- strain to pass dung- gets worse with each attempt. Occurs in cows (not calf) in paddy or shed.
3. “Gol –phulla” aka “Moh Bissony” (Honeycomb fan): swelling starts at chin- runs down brisket, feels soft and warm- not painful, animal doesn’t lose weight, animal never dies.
4. “Saboka”: Infection, wound between toes, sometimes maggots come, difficulty walking. Usually occurs after flood

**Can you teach me how you recognise the disease? Treat?** Prevent?

1. First 3 days- praying and burn incense- if not better in 3 days- call Dr- gives injection and tablets- animal gets well.
2. Local herbs- some from paddy and garden, nothing from jungle. Some are leaves, some roots- crush and give with black salt.
3. Pray, stroke neck- effective- better in 2 days. Must start praying within 24 hours, otherwisde animal may die (compare with above).
4. “Dhunna”- make Pooja with incense and prayer, if signs of saboka- give “dhunna” (tree sap- “

**How often do these problems occur?**

1. After flood 2016
2. Before flood 2016
3. After flood 2016
4. One ox continually has a wound.
5. 2014
6. Long time ago
7. See above
8. 2016

**What things limit how much your animals give?** ( provide for your household?)

**Young Animals**

Where do you keep your calves? Can we see please?

**How many of your cows/ buffs/ goats had a baby in the last year?**

2 cows, 2 calves

**Where are those baby animals now?**

**When a calf is born, can you teach me about the first day of its life?**

(When should it first drink milk (colostrum)? How much? What can you do to help it to be healthy? Who helps it?)

If dystocia- call Dr., no experienced men in this area.

Clean cow, clean calf, clean navel with coconut oil, watch that no birds peck at navel cord.

Feed cow- cabbage or any available veg, rice husk.

Observe carefully and check that calf is drinking.

Phaa Hoo: before calf drinks milk out 1L from cow using all 4 teats. Make Phaa Hoo sweets, make also a lucky string of Phaa Hoo beads and put round cow and calf neck. Calf drinks half after cow is milked. Cow is milked 1 hour after calf is born, calf first drinks at 1.5 hours old.

Concerned about diarrhoea so prevent calf from drinking “Not drinking first yellow milk prevents calf from having diarrhoea.”

**How much milk does an older calf drink and how often does it get it?** (Can you teach me how you do this? Who is responsible for this?)

Next milking after 10 days, calf is fully drinking for 10 days, cow and calf together, cow is grazed but not tied when she has young calf.

Milked once daily for first month, then twice daily. Separate cow and calf at 2am, in morning let calf drink for 3 minutes then milk cow for 1 – 1.5L, some milk left in udder for calf.

**Do your calves have any food apart from milk? Water?**( Where? How often? Who’s job?)

Start grazing at 1.5 months old.

Give water at noon and evening.

Self or mother does.

**How much milk does your family get each day from your animals?** (What do you do with it? When do you first milk a cow/ buff /goat for drinking?)

1 - 1.5L cow/day, sell at little

If milking twice daily- nearly 2L

I milk the cow

**Adult Animals**

**Can you show me where your animals rest in the day? At night?**

Shed night, paddy day

**How do your animals get food? What**? (Who (family member) is responsible for this? Can we meet them please? How much land do you have? Can we see it?) **Where do your animals drink? How often?**

Cooked food- veg, rice water, rice husk, salt

During flood- banana tree chopped, elephant grass, rice straw, weeds from wetland.

Water 3- 4 times daily in flood, other times twice daily.

Self and mother do.

**Did you vaccinate your animals last year? Why / why not?**

Yes. 10 years. Prevent disease- Hagoni, Kura patta, Pet fulla.

**Do you de-worm animals? How? Why/ why not?**

No.

“Pel- lou”: worms

“Nee- mok”: salt

**Do you do anything else to help your animals?**

Concentrate mixture (powder)- give to lactating cows to produce more milk. Cooked like rice husk, smells slightly like yeast. 200- 300g mixed with veg or rice husk once daily.

**You live close to the forest reserve. Does this affect you?**

Crop raiding- wild animals, elephant, rhino

Tiger killing cattle. 4 animals- 3 cows and 1 calf (3 year old). Last time May 2014, occurred in shed- saw tiger and scared tiger away. WWF/ Forest Dept compensation Rs4000 (WWF Rs2500, FD Rs1500) per cow. Took 6 months to pay, Rs20,000 total. Bought new cow.

New cow Rs 15k, Ox Rs 30K – 40K/ pair or >15K single.

**Animal health education**

**Is there anyone who can treat your animals?** (What would you like the vet doctor to know more about?)

**Would you like to learn more about animal health? Why?**

**What problems are there for people trying to learn about animal health in your village?** ( Why? Which ways of learning about animal health would be good for you (village meetings, classes, leaflets, radio broadcasts, internet information, etc)?)

**What changes could make your farm to more productive/ better?**

**What do you think is the future of this farm? (**Who will you pass your knowledge of farming on to?)

**Anything else you would like to tell us?**

**16 and 17**

**Village**: **Date**: 23/3/17 **Time**: 9.40 am

Interviewer: Andy Translator: Jadu

**Location/ who else is present / what else is happening?**

**Name**: (Husband) **Male/ female:** **Age:** 44

(wife) 38

(Friend) 55

**Your family and household**

**Who lives in your house?**

(Can we meet them please? What do they do? What do you want to do when grown up?)

**16**

Selfs

Daughter 18 BSc botany 2^nd^ semester

Son 16 HS 1^st^ year

Hopes children will get jobs- depends on what qualifications they get and what they want to do- never thought about what would happen if children did not want to farm later on

**17**

Wife

Son 23 Works for a company in Hyderabad- feels proud but is sad that he is not here

Daughter 28 Helps in house

**How is your family/ household supported?** (food, money, rent, etc).

**16**

Farming: Paddy, mustard, veg- toms, dhania, carrots, all kinds- sell veg in Bokahat

Business: sell (and transport on motor bike) supplies e.g. bags, candles in Bokahat

**17**

Farming: Paddy, mustard, veges- all kinds- eat and sell

**What animals does your family/ household have? Why are your animals important?** (Can we see your animals please? Why do you keep them? How do animals help to support your household? )

**16**

Cow: 1 milk

Calf: 1 sell, never keep

Goat: 1 Kid: 1 sell always, never eat

Duck: 1 Eat, sell, eggs- eat and more chickens

Chicken: 4 Eat, sell, eggs- eat and more chickens

Pigeon (“Para”): 50 Eat, sell, eggs only for more pigeons

Ox: 0

“We rent tractor and driver Rs300 / Bigha. Price of ox is too high and with other business there is no one to look after ox. Tractor is easier and can do more work than an ox in little time.

If conditions were good we could buy oxen, but it is difficult to get a man to work them. Oxen are very useful, but because of money and man problem we are not buying them.”

In Assam: 1 Bigha = 100ft x 100ft 4 Bigha = 1 acre

**What is a ‘good’ animal?** How do you know?

Physique, length, size.

If cattle are short then legs are small, if body is thin then body will become long.

If long legs and round body- this is very good.

Ox- hump should be big.

**How do you know if an animal is healthy?** How do you know if an animal is unhealthy?

Stop eating, body hot, body shaking, high temperature, lay down, bad mood.

**What diseases/ problems are important for your animals?**

*Friend arrives at this time, lady goes into house to work.*

1. “Kura- phatta”: wound in gap between toes, becomes larger, maggots form in wound. Happens after flood, starts with one animal and spreads to all in shed. “Mukot- ghahua”- wound in mouth, also in tongue.
2. “Becculia” aka “Dingi- phulla”: Swelling from chin, spreads down to chest. Unable to eat. Fluid inside swelling- can feel it. Some may die. May die if not treated. (dingi-phulla- 50% die) occurs in any season, one animal at a time. If stop eating die in 7 – 10 days, if survive become very thin- if they try to eat start coughing so don’t eat.
3. “Jor” (fever): Shaking, stop eating, drip saliva. Mostly occurs before rainy season. Sometime only one, not many.
4. “Basanta” aka “Bohonta”: swelling of ears, stop grazing, animal won’t stand in shed, always goes to shade. Dung very tight, fever signs. May die if falls down. If animals skin breaks- may survive. If skin breaks flies can lay eggs and maggots form. Occurs during flood when animals go to high areas and are in sun and rain all the time. During flood little food is available. Heat of sun causes problem, animal may break rope to get to shade. Not associated with skin colour- black animals never lay in the sunlight- not because of Bohonta. Animals are grazing normally before, during flood eat rice straw, rice husk and cooked food. Rice straw sometimes goes mouldy if water gets into straw. Eating ipomea causes diarrheoa, if animal eats more loses vision. We try to prevent animals from eating ipomea.
5. “Papora- dhora”: goat skin disease- rubs body against anything, very itchy, hair falls, skin rough and white colour with some boils. Spreads and can affect all (many?) goats, starts after flood and goes on after that. Get thin. Can affect dogs also. May affect calves also. Become well later but takes time.
6. “Letekua”: water from eye, eye goes white, may go blind. One or both eyes affected. Affects cows more than goats. One at a time, maybe two or three, worse if weather is hot. I don’t associate it with dust or flies.
7. “Sikora” (ticks): all animals have. See when big, gets on when small but we see when big. More in winter. If a cow has many sickora it may get thin and weak but do not die. Small sikora- white, big sikora mud colour (*grey?*) with no pattern or very thin stripes on body (*different species?*). If so many sikora may look like skin rash. Bigger (thumb sized!) sikora on pig.

Lantana – “Phutoo- gani”- not found here (*I saw close by*).

“Kukur” = dog

“Ou- koni” = flea

**Can you teach me how you recognise the disease? Treat?** Prevent?

1. Tie in mud or call Dr. No local (herbal) treatment. Different treatments from home to home.
2. Cut banana leaf (“Bhem- chol”- particular type) burn and mix ashes with salt- animal eats .and apply to wound (what wound?) for 2-3 days- effective. “Bhem – chol” aka “Chol-a-char” is good for humans also- eat with daal.
3. Dr injection, no local treatment.
4. If skin breaks internal heat is released and animal lives.
5. Wash with fish washing water, I think this works, also get Dr medicine. Animal always lives.
6. Wash eye with salt water- can be helpful (*Did not say is effective*). Herb “Dhrune” grows by river- crush leaf, mix with salt and wrap in a clean cloth, press and drip juice in eye- very effective- total cure. “Dhrune”- also used in people with sinus problems- very medicinal for humans in many ways- eat with daal or sabzi. Also givet o cow that is not eating- wrap in banana leaf, warm add salt- cow eats.
7. Wash cattle with tobacco water- effective.

**How often do these problems occur?**

1. November 2016
2. 2016
3. Not in my cows.
4. 2016 cow died
5. Not
6. Now happening (March)
7. Some now, all year problem, worse in November- December.

**What things limit how much your animals give?** ( provide for your household?)

**Young Animals**

Where do you keep your calves? Can we see please?

**How many of your cows/ buffs/ goats had a baby in the last year?**

1 cow had one calf

2 ox, 2 cow, 2 calf (1 born last year), 4 ducks.

**Where are those baby animals now?**

**When a calf is born, can you teach me about the first day of its life?**

(When should it first drink milk (colostrum)? How much? What can you do to help it to be healthy? Who helps it?)

Clean calf, help to stand, help to drink from mother- usually drinks after half an hour.

Phaa- ho: calf drinks first until full, then we milk, usually we get less than 1 litre, occasionally more than a litre if it is a very milky cow (17). We both do the same (16)(*Not sure of the veracity of this!*).

**How much milk does an older calf drink and how often does it get it?** (Can you teach me how you do this? Who is responsible for this?)

First milking at 10 -0 12 days- cow and calf together until then.

Separate calf at midnight, once calf is 3-4 months old- separate at 9 or 10 o’clock.

In morning calf drinks first for 10 minutes, then milk cow. We take less milk for the first month- 0.5L – then 1 – 1.5L daily after that. Milk once daily.

16- wife milks cow 17: husband and wife milk cow.

**Do your calves have any food apart from milk? Water?**( Where? How often? Who’s job?)

Both men same.

Calf eats food from 3- 4 months- eats cooked food with mother.

Calf starts to taste grass from 1 month. Some people milk the cow empty twice daily- then calf starts eating grass at 2 months- calf becomes more healthy and health of cow improves too.

Water 2 or 3 times daily

16: I am outside the village with business, my son and daughter work so my wife does all this kind of work.

17: Man and woman both do.

**How much milk does your family get each day from your animals?** (What do you do with it? When do you first milk a cow/ buff /goat for drinking?)

17: 0.5 – 1.5L daily / cow (depending on animal, stage of lactation) sell 1L /cow/ day =2L

16: ? (*wife tells husband*) drink it all, son drinks 0.5L daily, more if he can get it.

**Adult Animals**

**Can you show me where your animals rest in the day? At night?**

Day: paddy (“Patar”) –tie cow, calf free. Night: shed

Take to water lands (Kumud and Umesh)

**How do your animals get food? What**? (Who (family member) is responsible for this? Can we meet them please? How much land do you have? Can we see it?) **Where do your animals drink? How often?**

17: Grazing, rice straw (little given every day) cooked food- rice husk, pumpkin, banana, brinjal, bottle gourd, carrot

16: Rice husk, veges, cauli, aloo; rice straw

Flood time: keep most of straw for flood time, banana tree, if Forest Dept let us cut elephant grass feed that and weeds from water lands (“Dol- ghah” = elephant grass)

“Gha” = grass

**Did you vaccinate your animals last year? Why / why not?**

17: No. Did only once- cow’s neck swelled but became well again, after that scared of vaccination

**Do you de-worm animals? How? Why/ why not?**

“Pel- lou” = worms. I never see any kind of worms in my cow dung so I never give medicine. I always give my animals salt and perhaps that is why I never see worms.

**Do you do anything else to help your animals?**

16: Keep shed clean every day. I light incense in there every day. Usually I wash my cattle, I wash my oxen after they cultivate, I wash my cows once a month. Mosquitos nest in dung.

17: “I take very good care of my cattle. If I see any kura phata in village I wash all my animals in hot water with salt or KMnSo_4_. Because of this I have not had Kura- phata in my cows for 8 years. I think only I do this, but sometimes I advise other people to do this as well. If you prevent in early days then disease does not have to spread to all animals. I advise other people to do this. If you cannot find KMSO_4_ then you must use salt.”

**You live close to the forest reserve. Does this affect you?**

17: “We mostly rely on crops. Crop raiding by elephants and wild boar is our most important problem. This is the main problem and nothing else. Sometimes elephants eat banana trees. It is in their nature, it is not a big problem. Forest guards do come to help us with thus problem. TCF have helped us providing tungi and torches. These have helped us to scare off elephants, we are very grateful to them.”

**Animal health education**

**Is there anyone who can treat your animals?** (What would you like the vet doctor to know more about?)

17: Dr comes 3.5km. He comes mostly when we call. He is a good Dr and I like him.”

**Would you like to learn more about animal health? Why?**

17: “I would like to learn more. It would be helpful to me.”

**What problems are there for people trying to learn about animal health in your village?** ( Why? Which ways of learning about animal health would be good for you (village meetings, classes, leaflets, radio broadcasts, internet information, etc)?)

17: “I know how to read and write in Assamese. If I got some posters and pamphlets I can learn from these. If photos are provided I can learn from these too but it should be in Asssamese. Practical training is also very useful because people can show me and I can learn by doing.”

**What changes could make your farm to more productive/ better?**

17: “I want to buy some high breed cows that can make more milk. I can afford to feed Jerseys which are cross breed with local cows, they need less food than pure bred cows.

**What do you think is the future of this farm? (**Who will you pass your knowledge of farming on to?)

16: The next generation

**Anything else you would like to tell us?**

*Umesh’s wife arrives*

“We are very grateful because you came and you teach us important things. We are very eargerly waiting to see you again.”

Umesh’s wife: “Nowadays the village is improving a little because we have a road to come here.”

“Usually we are doing crops in the winter time. The flood washes out every crop. Now we are doing the (un)seasonal crop because we have the water motors (oumps) and things are improvinhg bit by bit. Because of the flood and the crop raiding pattern we changed. Now the raiding pattern is changing too.”

Umesh

8.5 bihars: 2.5 Govt (farmed for many years), 6 rented + 3 bihars mustard.

If not raided- 3 bihars is enough to support us. I get 4 -5 quintals (1 quintal = 100kg) per bihar because I use less fertiliser and pesticide. Elephant takes more than 50% of crop. 2016 seasonal crop I got only 200kg from 4 bihars due to elephant raiding.

3 types of crop:

Hali: June – Nov/December

Bow: June – Nov / December (different type of rice)

Bodo- khati (irrigated crop): January – June (harvest before flood)

Seasonal crop- start little plants before flood, plant out after flood while rains still sufficient

“Khati” = cultivation

**17**

**Village**: DFP **Date**: 23/3/17 **Time**: 1pm

Interviewer: Andy Translator: Jadu

**Location/ who else is present / what else is happening?**

In house, small (3?) daughter and mother in law present, men outside in yard.

**Name**: Lady and Mum in law **~~Male~~/ female:** **Age:** 25 + don’t know ( 50+)

**Your family and household**

**Who lives in your house?**

(Can we meet them please? What do they do? What do you want to do when grown up?)

Self

Husband 28 carpenter

Daughter 3

Sister in law 32

Brother in law 34 can do many jobs for money (handyman)

Brother in law 27 carpenter unmarried

Father in law

Mother in law

**How is your family/ household supported?** (food, money, rent, etc).

1. Carpentering
2. Farming- paddy (home), mustard (sell), veg- home and selling
3. Weaving- wants to expand, get sewing machines and get women working to make money through tailoring business.

**What animals does your family/ household have? Why are your animals important?** (Can we see your animals please? Why do you keep them? How do animals help to support your household? )

Cow: 2 milk, calves

Calf: 1 keep healthy female for home, always sell male after harvest approx. 1 – 1.5 y/o

Goat: 1 sell- castrate male (Dr does castration)

Duck: 5 selling, eating, eggs

Chicken: 30+ selling, eating, eggs

Ox: 0 Sold because of money crisis. Normally sell ox when old

“Use rented tractor. Father had a stroke, had to sell ox. Want to buy oxen now, then we won’t have to pay tractor. Ox is best, you can do whatever/whenever you want.”

**What is a ‘good’ animal?** How do you know?

Colour, health, physique. Ears erect, eyes (you know if animal is healthy by looking, legs- muscular, hoof- should be pointy, tight shape, upright, small gap.

“Colour- red and black mix, no white. White is not so good, not suitable for this home”

Cow: strong backside, if cow and calf, then look at calf.

“To buy (my father in laws’) sons all go together. Sometimes an experienced man from the village goes with them if available.”

“Saboo” = eyes

**How do you know if an animal is healthy?** How do you know if an animal is unhealthy?

Sad mood, stops eating, lays down, doesn’t want to go to graze.

**What diseases/ problems are important for your animals?**

1. “Bohonta”: sad mood, skin falls off in 4 - 5 days. Worse in summer and start of rainy season. One animal then occurs in others. Ay die, lose weight. Disease is worse in rainy season and less survive.
2. “Jor”: Occurs at any time. Sad mood, stop eating, lay down a lot, won’t walk. One animal at a time.
3. “Pet phulla”: Big belly. Cow sad, laying down a lot, won’t eat- belly is big, makes some bad noises, can’t pass dung or urine. I don’t know about breathing (*response to question*). Some die, some become well.
4. “Papora dhora”: Winter season, only goats affected, spreads between animals. Hair falls, skin is rough and white, can spread to whole body.

**Can you teach me how you recognise the disease? Treat?** Prevent?

1. “Dhuna” (powdered tree sap)- feed powder and burn incense in sheds. Faith healing for 3 days- if not better then call Dr.
2. Fever gets well on its own, give good food. We don’t call Dr.
3. Get mud from a rat hole and throw at cow.
4. Paste of turmeric and mustard- effective. If not better- cover with … hydroxide- effective.

**How often do these problems occur?**

1. After flood 2016
2. After flood 2016
3. 2013
4. 2016

**What things limit how much your animals give?** ( provide for your household?)

Lack of fodder, especially during flood.

**Young Animals**

Where do you keep your calves? Can we see please?

**How many of your cows/ buffs/ goats had a baby in the last year?**

2 cows, both calved

**Where are those baby animals now?**

1 here, 1 sold (we use calves as savings)

**When a calf is born, can you teach me about the first day of its life?**

(When should it first drink milk (colostrum)? How much? What can you do to help it to be healthy? Who helps it?)

Check navel, clean calf, check for wounds/ maggots ( do nothing if none found), if maggots present put Himax^TM^ or tobacco, may body bandage to colour.

Feed cow banana leaves (gol- phat) and warm water, especially if placenta did not pass.

Clean and tie rice straw in calf’s mouth (for maximum 30 minutes), calf then drinks for 10 – 30 minutes, calf is usually drinking 20- 30 minutes after born, then wash udder and milk out phaa-hoo, amount of phaa-hoo gained varies with udder size, usually about 1 litre.

**How much milk does an older calf drink and how often does it get it?** (Can you teach me how you do this? Who is responsible for this?)

Next milking at 10 -11 days, cow and calf together all the time until then. Milk cow once daily- separate calf from cow in the morning- milk cow 3 -4 hours later- calf drinks for 5 minutes- then milk. Get 1 litre / cow per day. (2 cows therefore 2 litres)

**Do your calves have any food apart from milk? Water?**( Where? How often? Who’s job?)

Start eating at 2 – 3 months old. Grazing plus cooked food with mother.

Take water with mother cow.

Mother in law is main cow carer, she milks and takes water. Other family members help if available.

**How much milk does your family get each day from your animals?** (What do you do with it? When do you first milk a cow/ buff /goat for drinking?)

1 L/ cow / day = 2L usually use one and sell one.

**Adult Animals**

**Can you show me where your animals rest in the day? At night?**

Paddy in day- tie cow, calf loose, shed at night

**How do your animals get food? What**? (Who (family member) is responsible for this? Can we meet them please? How much land do you have? Can we see it?) **Where do your animals drink? How often?**

Water 3x daily- mother in law does

Cooked food: chopped banana tree and veg, occasionally cow food mix.

In flood- rice straw, banana tree and leaves.

**Did you vaccinate your animals last year? Why / why not?**

Yes. For at least 5 years. Mother in law knows why, young lady also knows why- it’s about preventing Dz- especially saboka.

**Do you de-worm animals? How? Why/ why not?**

Give medicine- adult cows at 1 year. Never to calf or goats.

**Do you do anything else to help your animals?**

Vitamins sometimes.

**You live close to the forest reserve. Does this affect you?**

**Animal health education**

**Is there anyone who can treat your animals?** (What would you like the vet doctor to know more about?)

Dr- Saboo- 4km away. He comes when called. I like the Dr.

**Would you like to learn more about animal health? Why?**

Yes. If I know more about cattle I can produce more.

**What problems are there for people trying to learn about animal health in your village?** ( Why? Which ways of learning about animal health would be good for you (village meetings, classes, leaflets, radio broadcasts, internet information, etc)?)

“If someone explains in a good way I can learn from meetings. Also I can learn from practicals.” (young lady)

“Yes, I can learn from leaflets, but day by day my eyesight is dropping. Dr told me to wear glasses but I am not doing.” (old lady)

**What changes could make your farm to more productive/ better?**

“Buy high quality hybrid cows to increase production. I think I can produce for most of the year, but there will not be sufficient fodder in flood time. That is why I am not buying (cows).”

“I think a lot, that’s why my hair is going white.” (old lady)

**What do you think is the future of this farm? (**Who will you pass your knowledge of farming on to?)

“Son, and then I don’t know, I can’t predict the future.”

**Anything else you would like to tell us?**

“I am very tranquil, but I don’t understand everything you said.” (old lady)

“I do want to learn more about animal health. I am very grateful to meet you, I am hoping to see you again in the future.”
