# Supplementary material for: Animal health perceptions and challenges among smallholder farmers around Kaziranga National Park, Assam, India: A study using participatory epidemiological techniques
Source: PLoS One. 2020 Sep 24;15(9):e0237902. doi: 10.1371/journal.pone.0237902 (PMC7513994; doi:10.1371/journal.pone.0237902)
Supplement: S1 Table — (DOCX) [file pone.0237902.s004.docx]

# S1 Table

### **Household livelihood steams of interviewees**

The number of interviewees (n=18) reporting one or more household members pursuing various income streams is shown in the table below

| Income stream | Number of households with one or more member undertaking activity |
| --- | --- |
| Farming | 18 |
| Weaving | 13 |
| Carpentry | 5 |
| Daily labour | 4 |
| Small shopkeeper | 3 |
| Trading business | 3 |
| Driver | 2 |
| Primary school teacher | 2 |
| Local government representative | 1 |
| Military salary | 1 |
